# Guts of the Urban Ecosystem: Microbial Ecology of Sewer Infrastructure

**DOI:** 10.1128/msystems.00118-22

**Published:** 2022-06-28

**Authors:** Adélaïde Roguet, Ryan J. Newton, A. Murat Eren, Sandra L. McLellan

**Affiliations:** a School of Freshwater Sciences, University of Wisconsin-Milwaukee, Milwaukee, Wisconsin, USA; b Helmholtz Institute for Functional Marine Biodiversity, Oldenburg, Germany; c Josephine Bay Paul Center, Marine Biological Laboratory, Woods Hole, Massachusetts, USA; University of California San Diego

**Keywords:** *Acinetobacter*, *Arcobacter*, antibiotic resistance, metagenomics, microbial communities, sewer infrastructure

## Abstract

Microbes have inhabited the oceans and soils for millions of years and are uniquely adapted to their habitat. In contrast, sewer infrastructure in modern cities dates back only ~150 years. Sewer pipes transport human waste and provide a view into public health, but the resident organisms that likely modulate these features are relatively unexplored. Here, we show that the bacterial assemblages sequenced from untreated wastewater in 71 U.S. cities were highly coherent at a fine sequence level, suggesting that urban infrastructure separated by great spatial distances can give rise to strikingly similar communities. Within the overall microbial community structure, temperature had a discernible impact on the distribution patterns of closely related amplicon sequence variants, resulting in warm and cold ecotypes. Two bacterial genera were dominant in most cities regardless of their size or geographic location; on average, *Arcobacter* accounted for 11% and Acinetobacter 10% of the entire community. Metagenomic analysis of six cities revealed these highly abundant resident organisms carry clinically important antibiotic resistant genes bla_CTX-M_, bla_OXA_, and bla_TEM_. In contrast, human fecal bacteria account for only ~13% of the community; therefore, antibiotic resistance gene inputs from human sources to the sewer system could be comparatively small, which will impact measurement capabilities when monitoring human populations using wastewater. With growing awareness of the metabolic potential of microbes within these vast networks of pipes and the ability to examine the health of human populations, it is timely to increase our understanding of the ecology of these systems.

**IMPORTANCE** Sewer infrastructure is a relatively new habitat comprised of thousands of kilometers of pipes beneath cities. These wastewater conveyance systems contain large reservoirs of microbial biomass with a wide range of metabolic potential and are significant reservoirs of antibiotic resistant organisms; however, we lack an adequate understanding of the ecology or activity of these communities beyond wastewater treatment plants. The striking coherence of the sewer microbiome across the United States demonstrates that the sewer environment is highly selective for a particular microbial community composition. Therefore, results from more in-depth studies or proven engineering controls in one system could be extrapolated more broadly. Understanding the complex ecology of sewer infrastructure is critical for not only improving our ability to treat human waste and increasing the sustainability of our cities but also to create scalable and effective sewage microbial observatories, which are inevitable investments of the future to monitor health in human populations.

## INTRODUCTION

Sewer systems perform an essential role in collecting and transporting wastewater in cities, which prevents the rapid spread of diseases caused by human enteric pathogens. Sewer systems are often extensive but unseen, as they encompass thousands of kilometers of conveyance pipes beneath cities (1). This unseen system contains a diverse set of microorganisms that are continuously transported to wastewater treatment facilities. Significant progress has been made in understanding the composition and activity of the microorganisms involved in and removed by treatment processes at wastewater treatment plants (WWTPs), but the biological and ecological processes in the conveyance systems are not well understood. For example, microbes in WWTPs have received considerable attention for their ability to remove excess primary nutrients (carbon, nitrogen, and phosphorus) and degrade toxic compounds from wastewater before it is discharged into the environment (2–5). However, the resident sewer microbiome has not been explored in depth for its metabolic potential for pretreatment of waste within the vast network of pipes.

Sanitary sewer pipes have three main microbial habitats: wastewater, biofilm, and sediment, each hosting a distinct and considerably diverse microbial community (6–8) involved in various forms of carbon, sulfur, and nitrogen metabolism. To date, sewer bacterial assemblages have been studied primarily in biofilms with a focus on detrimental effects, such as creating nuisance odors and contributing to pipe corrosion from H_2_S or H_2_SO_4_ emission (9). A growing number of studies also indicate that sewer microorganisms can transform and/or remove pollutants (5, 10, 11), making the sewer network a full-fledged stage in water treatment.

Sewer conveyance systems are a habitat that is aqueous, dark, and with high levels of nutrients. Gravity sewers, which are common in most cities, can be aerobic but become anaerobic or microaerophilic depending on flow, turbulence, organic matter, and temperature (12). Despite these strong fluctuating conditions, modern sanitary sewer systems hold a surprisingly coherent and reproducible taxonomic composition worldwide (13). Upstream in the sewer network, the bacterial community appears to contain a higher proportion of fecal-associated bacteria (~35%) compared with downstream (~10%), where it is dominated by nonfecal-associated taxa (14, 15), in particular, (alphabetic order) *Acidovorax*, Acinetobacter, *Aeromonas*, *Arcobacter*, *Cloacibacterium*, or *Trichococcus* (16–19). These organisms dominate regardless if the sewer system is separated (i.e., carries only sanitary sewer) or combined (i.e., conveys a mix of wastewater and stormwater during rain events) (18, 20). A better understanding of the sewer microbial composition, including identifying universally present members, and understanding how the microorganisms interact with each other could lead to common engineered controls to enhance beneficial metabolism or reduce detrimental effects. Such controls could also influence the efficiency of biological treatment at WWTPs, as it has been shown that the microbial community in raw wastewater shapes those in WWTPs (21, 22).

Untreated wastewater entering the WWTP contains an abundance of antibiotic resistant bacteria and associated resistance-conferring genes. Antibiotic resistance genes occur in pathogenic but also nonpathogenic bacterial populations in these systems (23). High-abundance resident pipe bacteria include Acinetobacter spp., *Arcobacter* spp., and *Aeromonas* spp. known to carry multidrug resistance (24–27). There is growing interest in using wastewater for population health surveillance; therefore, it is important to understand the fraction (human or resident) of the community contributing to antibiotic resistance profiles observed in wastewater and the potential for various microorganisms to propagate or transfer resistance traits within these systems.

In natural systems, physical separation and unique local environmental conditions lead to the diversification of organisms (28). Although cities are separated islands in a landscape, the engineered habitat they host leads to similar communities across systems. Therefore, it may be possible to identify and promote universally adapted organisms with beneficial functions through further engineering measures (29). Also, a deeper understanding of the ecology of the sewer ecosystems can contribute to the development of baseline metrics of microbial community signatures to better identify concerns of public health, especially against the background of antibiotic resistance genes that occur naturally. Such insights would allow for the creation of more universal sewage pollution tracking assays, including those that are more sensitive than tracking microbes from human feces. Here we use microbial community structures in a large data set of 71 U.S. cities and metagenomes of six cities to provide insight into the distribution of the microorganisms in this unique environment that represents the “gut” of every city.

## RESULTS

**FIG 1:**
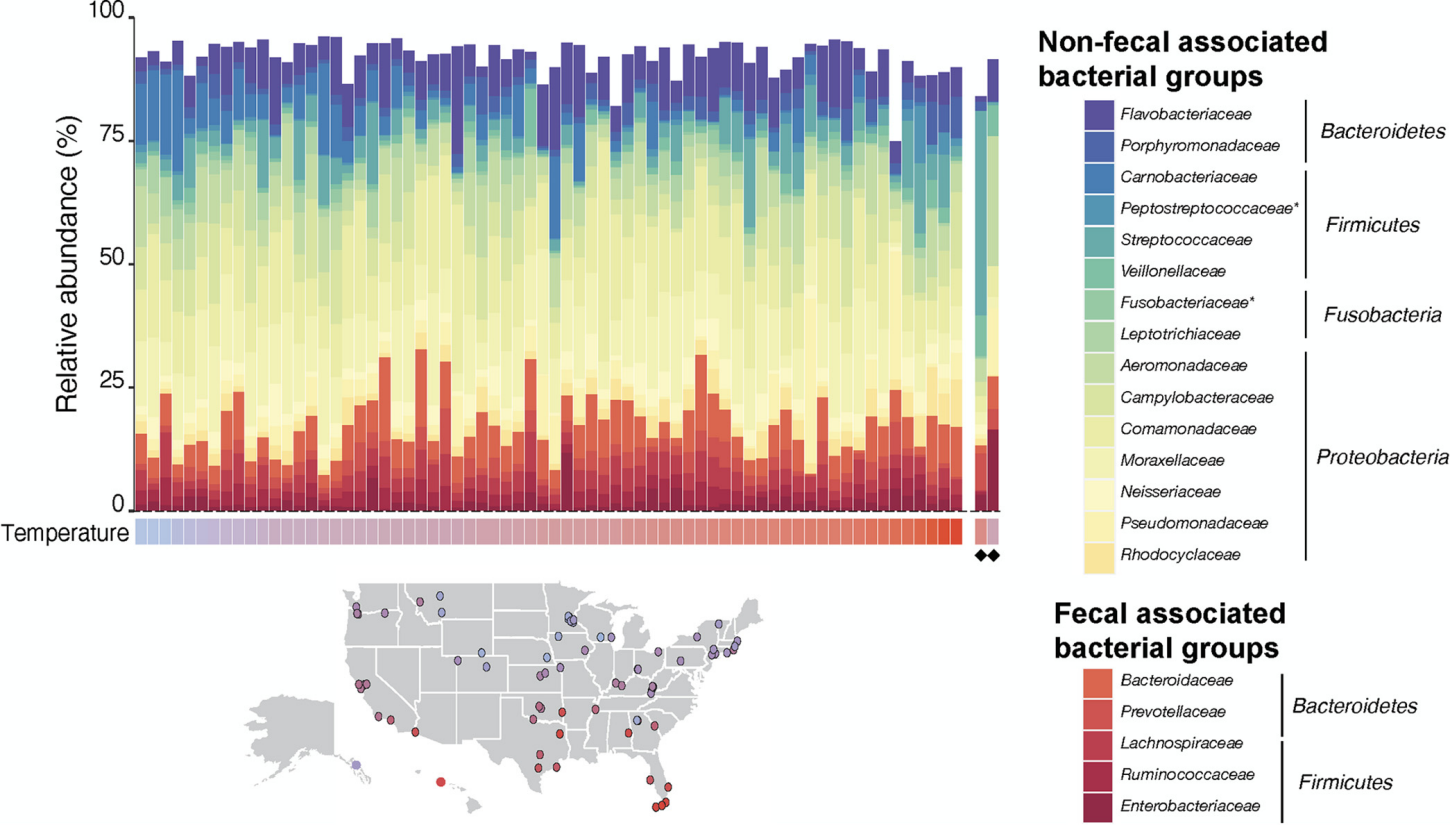
Relative abundance of the top 20 bacterial families across 71 influent samples sorted by the collection date average air temperature, January samples (ordered left to right). The temperature ranges from −17 to 19°C. Asterisks indicate families with an average relative abundance of greater than 1% of the total bacterial community across the 211 domestic sewage samples. Black diamonds indicate industrial wastewaters.

Bacterial communities from 215 domestic sewage samples across 71 U.S. cities and 1 city in Spain (Fig. 1; Data Set S1 in supplemental material) were characterized by sequencing the V4–V5 variable regions of the 16S rRNA gene. Minimum entropy decomposition analysis was applied to 11,924,709 sequences, resulting in 1,893 amplicon sequence variants (ASVs) representing 96% of the initial sequence data set.

10.1128/msystems.00118-22.10 DATA SET S1 List of ASV sequences and meta data for samplesData Set S1, XLSX file, 5.2 MB. Copyright © 2022 Roguet et al. 2022 Roguet et al. https://creativecommons.org/licenses/by/4.0/ This content is distributed under the terms of the Creative Commons Attribution 4.0 International license.

### Reproducibility and coherence of microbial communities in sewer infrastructure.

**FIG 2:**
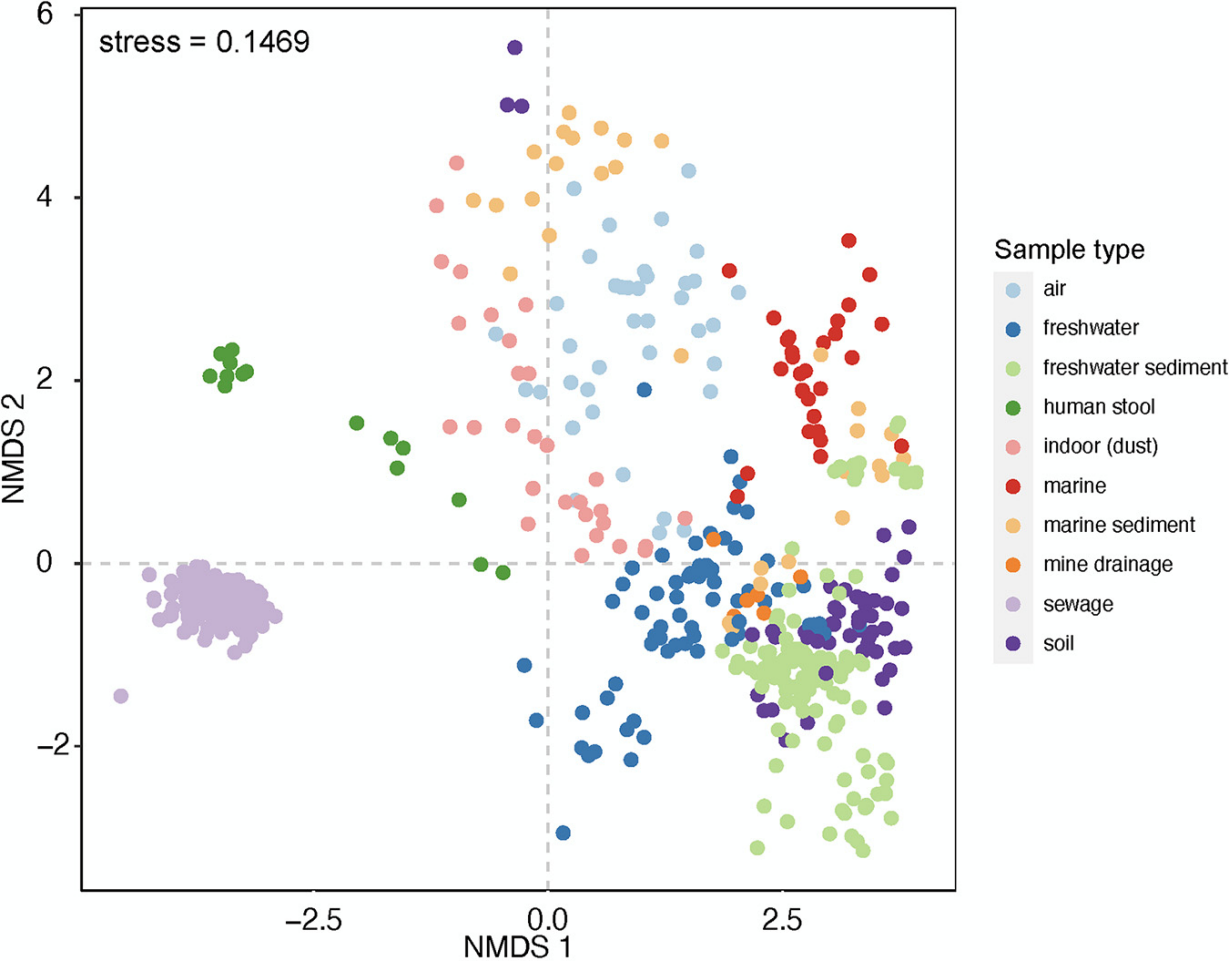
Nonmetric multidimensional scaling analysis of microbial communities in distinct aquatic and terrestrial biomes.

We observed a coherent and reproducible bacterial taxonomic composition among 71 U.S. city sewer conveyance system samples regardless of system size or geographic location (Fig. 1). The sewer microbiome was dominated by *Proteobacteria* (55.94 ± 9.78%, means ± SD), *Bacteroidetes* (24.28 ± 9.49%), and *Firmicutes* (16.10 ± 7.42%). There were only 18 bacterial families with an abundance of more than 1% of the total community (Fig. 1). These families were shared across all U.S. cities and the sample from Spain. The 18 families made up 92%, on average, of the 16S rRNA gene sequences in a sample. Although sewer systems receive significant microbial inputs from other microbial systems (e.g., human fecal microbiome, soil, groundwater), the bacterial community structure in sewage did not resemble any of the communities commonly found in these other ecosystems (Fig. 2).

Although overall bacterial community composition was similar across cities, on average individual cities had a more consistent bacterial assemblage among the three sampling dates than comparisons across cities (average Bray-Curtis dissimilarity index of 45% versus 62%, respectively; Fig. S1). Four samples with a high amount of industrial waste (estimated from 60% to 100% of load) had a distinct community structure (Fig. S2). These cities were the only ones estimating that industrial waste contributions accounted for at least 45% of the input to their sewer systems.

10.1128/msystems.00118-22.1 FIG S1 Distribution of Bray-Curtis dissimilarity indices of the sewer microbiome within and between 72 cities. “Intra-city” shows the pair-wise comparisons of the bacterial community of a wastewater treatment plant across the three sampling campaigns (January, May, and August). “Inter-city” shows the pair-wise comparisons between cities all campaigns combined (all months) or for a given sampling campaign (January, May, or August). Means are symbolized by the horizontal bars. ***P < 0.001 (Wilcoxon test). Download FIG S1, PDF file, 0.9 MB. Copyright © 2022 Roguet et al. 2022 Roguet et al. https://creativecommons.org/licenses/by/4.0/ This content is distributed under the terms of the Creative Commons Attribution 4.0 International license.

10.1128/msystems.00118-22.2 FIG S2 Nonmetric multidimensional scaling analysis of sewer microbial communities (a) from 71 U.S. cities and one Spain city (*n* = 215). Shapes indicate the sampling period campaign: January (triangle), May (square), or August (circle). Colors display air temperature gradient from lowest (−17°C, blue) to highest (28°C, red) temperatures. The four industrial sewage samples are indicated by a black border. Download FIG S2, PDF file, 0.1 MB. Copyright © 2022 Roguet et al. 2022 Roguet et al. https://creativecommons.org/licenses/by/4.0/ This content is distributed under the terms of the Creative Commons Attribution 4.0 International license.

### Temperature-driven patterns in sewer microbiome structure.

**FIG 3:**
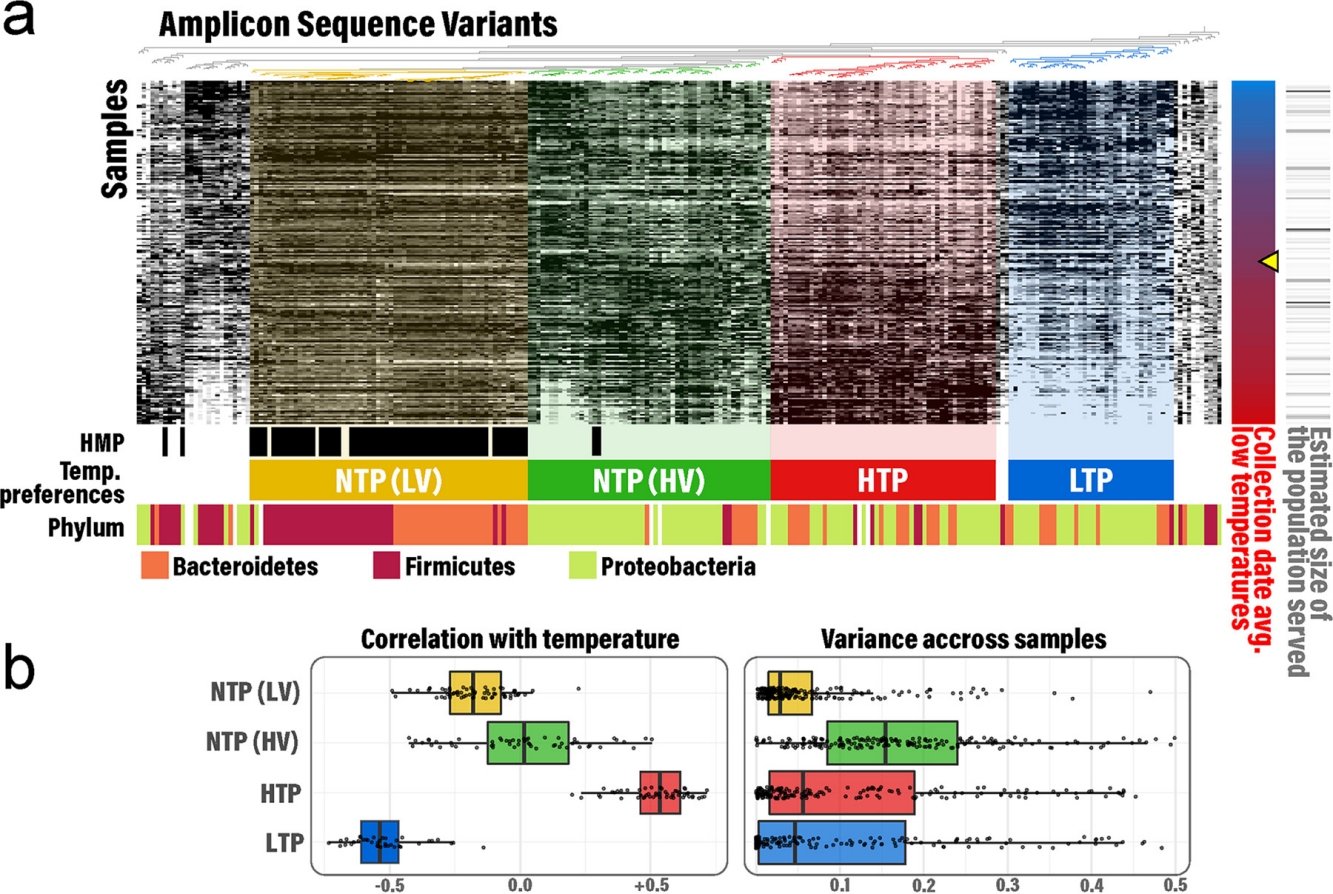
Bacterial community patterns within the 211 sewage samples. (a) The heatmap shows the relative abundance of the 250 most abundant amplicon sequence variants (ASVs) (white = low relative abundance; black = high relative abundance). Samples are sorted by average air temperature during sampling period from the coldest (blue, −17°C) to the warmest cities (red, +28°C). The estimated size of human population served by the wastewater treatment facilities is depicted on the right panel with the smallest population displayed in white (960 inhabitants) and largest population displayed in black (2,000,000 inhabitants). ASVs were clustered using the UPGMA algorithm based on the Bray-Curtis dissimilarity matrix. Four distinct patterns were visible, including ASVs with no-temperature preference and low-abundance variance across samples (NTP-LV), no-temperature preference and high-abundance variance across samples (NTP-HV), high-temperature preference (HTP), and low-temperature preference (LTP). The yellow cursor indicates the temperature threshold (10°C) at which the HTP and LTP community assemblages shifted significantly. (b) Distribution of the Spearman correlation between the ASV proportions and temperature, and the variance of the ASV proportions across the samples for each of the four temperature profiles. NMDS, nonmetric multidimensional scaling.

Underlying the consistency of the bacterial community structure in wastewater influent, distinct organism (based on ASVs) abundance patterns were observed across the cities investigated (Fig. 3). Air temperature best explained the ASV abundance patterns among the factors tested (redundancy analysis; Fig. S3). The 250 most abundant ASVs separated into three coherent abundance pattern bins, which we termed: no (NTP)-, high (HTP)-, and low (LTP)-temperature preference (Fig. 3). Using random forest regression, we determined that an air temperature of 10°C (9.7°C and 10.3°C for HTP and LTP, respectively) was the breakpoint between organisms at higher abundances in the LTP versus HTP bins. This measured air temperature corresponded to an estimated below-ground temperature (1-meter deep) between 12 and 15°C (see Materials and Methods). The temperature-based ecological preference was highlighted because the communities from warm cities were more similar to each other than they were to the communities from cold cities (Fig. S2). In addition, assemblages from cold cities were more similar to each other than those from warm cities. A larger shift was observed in the bacterial community composition between sampling periods for cities with a larger change in temperature between their winter and summer samples (Fig. S4).

10.1128/msystems.00118-22.3 FIG S3 (a) Variation partitioning analysis of the bacterial community structure of the top 250 amplicon sequence variants (ASVs) between spatial, environmental, population, and wastewater treatment plant (WWTP) factors. (b) The separate significance of each marginal constraint (i.e., spatial and environmental factors) was evaluated with an ANOVA-like permutation test (using the settings step = 1,000 and perm.max = 1,000) on redundancy analysis (RDA) testing these three factors only. Download FIG S3, PDF file, 0.1 MB. Copyright © 2022 Roguet et al. 2022 Roguet et al. https://creativecommons.org/licenses/by/4.0/ This content is distributed under the terms of the Creative Commons Attribution 4.0 International license.

10.1128/msystems.00118-22.4 FIG S4 Bray-Curtis dissimilarity indices of the sewer microbiome according to the maximum difference of temperature (temperature delta) between Winter (January) and Summer (August). Pair-wise comparisons within the same wastewater treatment plant between samples collected in May and August. Colors display air temperature (averaged among all sampling dates) gradient from lowest (−3°C, blue) to highest (23°C, red) temperatures. Groups sharing the same letter are not significantly different [ANOVA, *F*(4) = 3.218, *P* < 0.0179, (log10-transformed data), Tukey-adjusted, alpha = 0.05]. Download FIG S4, PDF file, 0.09 MB. Copyright © 2022 Roguet et al. 2022 Roguet et al. https://creativecommons.org/licenses/by/4.0/ This content is distributed under the terms of the Creative Commons Attribution 4.0 International license.

The NTP bin was further divided into two perceptible organism abundance patterns, ASVs having low (LV)- or high (HV)-abundance variation across samples. The NTP-LV ASVs, representing 25% of the top 250 ASVs, had remarkably consistent abundance across all sewer samples and were mostly assigned to the phyla *Bacteroidetes* and *Firmicutes*. Nearly all the ASVs in the NTP-LV bin had 100% sequence identity with V3–V5 amplicon sequences in the Human Microbiome Project human stool data set, suggesting that they represent a human fecal signature in wastewater. In contrast, the remaining 75% of the bacterial assemblage was assigned mainly to *Proteobacteria* and had much more variable abundance patterns across samples. Very few of these sequences matched those from the Human Microbiome Project human stool data set (Fig. 3).

We also examined the relationships among the top 250 ASVs by performing a co-occurrence network analysis (Fig. S5) for each of the three sampling campaigns (January, May, and August). We found that the human fecal-associated ASVs (NTP-LV) were highly interconnected, reflecting a strong cohesive co-occurrence pattern within this group. Very few of these ASVs had a network connection with a non-NTP-LV ASV, which indicates the abundance patterns of these ASVs are largely uncoupled from the community majority and do not respond to the dominant drivers of community composition change in these data. Moreover, these two modules were mainly split according to the phylogenetic origin of the ASVs, *Firmicutes* versus *Bacteroidetes*. This distinction could reflect a difference in physiology or decay between the two taxonomic groups (e.g., Gram-positive vs Gram-negative) within sewage conveyance systems.

10.1128/msystems.00118-22.5 FIG S5 Network analysis showing co-occurrence and modular patterns of the top dominant sewer ASVs for the January (a), May (b), and August (c) sampling campaigns. Node colors display the phyla, while the node's border colors indicate the temperature preferences (defined in Fig. 3 and S6n). ASVs with a prevalence > 80% and a maximum relative abundance of 10% are listed in black. Arrows indicate the nodes classified as module hubs (Z_i_ > 2.5 and P_i_ < 0.6). All the other nodes were described as peripheral (Z_i_ < 2.5 and P_i_ < 0.6). Solid and dashed edges represent positive and negative correlations, respectively. (d) Topological properties description of the networks. (e) The number of connections shared between the temperature preference groups. Bold displays positive correlations. Download FIG S5, PDF file, 1.1 MB. Copyright © 2022 Roguet et al. 2022 Roguet et al. https://creativecommons.org/licenses/by/4.0/ This content is distributed under the terms of the Creative Commons Attribution 4.0 International license.

Consistent with our cluster analysis, the network analysis also identified a major co-occurrence module consisting of HTP and LTP ASVs. This module was recreated in each of the three sampling months, a further indication that geographic factors influence the community composition. Among the 27 ASVs we categorized as dominant (Fig. S7), four were classified as module hubs, i.e., they had a high within-module connectivity (Z_i_). These ASVs included oligo_06371 (*Arcobacter*), oligo_07868 (*Acidovorax*), oligo_00948 (*Acetoanaerobium*), and oligo_08466 (*Cloacibacterium*). Furthermore, within this module, the LTP ASVs and HTP ASVs had the highest number of connections with ASVs with the same temperature preference classification.

10.1128/msystems.00118-22.7 FIG S7 Nonmetric multidimensional scaling analysis of the top 250 ASVs in sewer systems (*n* = 211). Dot colors display the temperature preferences determined in Fig. 3. Nonclassified ASVs are symbolized in gray. Background colors indicate the final classification of the nonclassified ASVs in Fig. 3. Abbreviations: no (NTP)-, high (HTP)-, or low- (LTP)-temperature preferences. Download FIG S7, PDF file, 0.2 MB. Copyright © 2022 Roguet et al. 2022 Roguet et al. https://creativecommons.org/licenses/by/4.0/ This content is distributed under the terms of the Creative Commons Attribution 4.0 International license.

### Dominance of nonfecal-derived genera in sewer microbiome.

**FIG 4:**
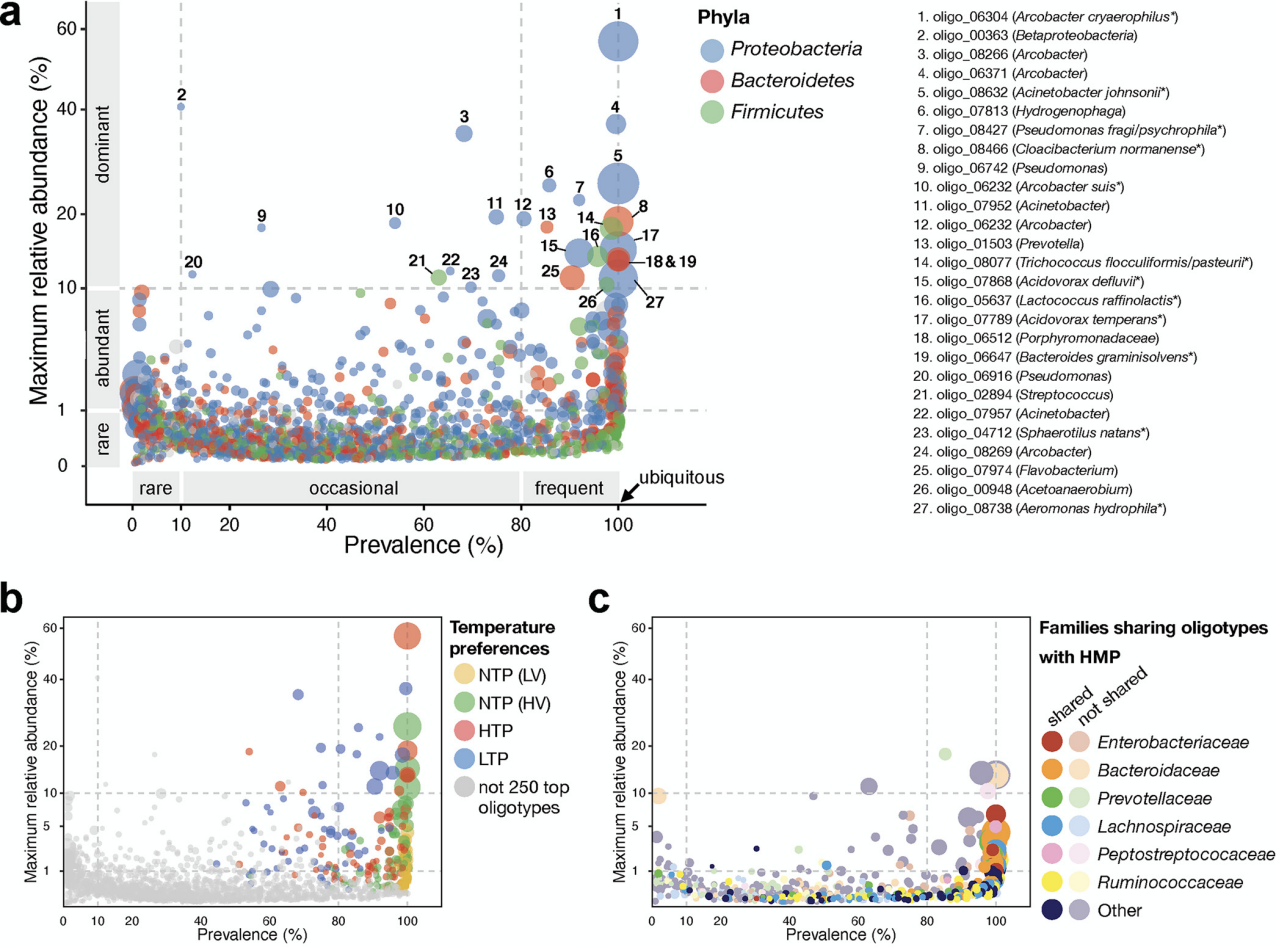
Maximum relative abundance of each ASV as a function of the prevalence of detection across the 211 sewage samples. (a) ASVs are color-coded by phylum. Asterisks indicate species with 100% match to sequences deposited in NCBI. (b) ASVs are color-coded by temperature preference patterns (see Fig. 3 and Fig. S7). (c) Families sharing ASV(s) with the Human Microbiome Project (HMP) stool data set. Symbol size corresponds to the median proportion of sequences for each ASV (when detected). The four sewage samples dominated by industrial waste were not included in this analysis.

Among the 1,893 ASVs in the sewer microbiome, 27 were considered dominant as they had a maximum relative abundance of at least 10% (Figure 4a). The top 10 genera were *Arcobacter* (with an average relative abundance of 11%), Acinetobacter (10%), *Bacteroides* (7%), *Acidovorax* (6%), *Cloacibacterium* (5%), *Aeromonas* (4%), *Flavobacterium* (4%), *Trichococcus* (2%), Pseudomonas (2%), and *Prevotella* (3%). These genera all had at least one ASV detected in ≥80% of sewage samples. Only seven ASVs were found in all sewer systems investigated. Among them, five shared 100% identity with species deposited in National Center for Biotechnology Information (NCBI): Arcobacter cryaerophilus (oligo_06304) the most abundant ASV (not detected in the 100% industrial sewage samples), Acinetobacter johnsonii (oligo_08632), Aeromonas hydrophila (oligo_08738), Bacteroides graminisolvens (oligo_06647), and Cloacibacterium normanense (oligo_08466). All have been previously isolated from sewage (24, 30–33).

**FIG 5:**
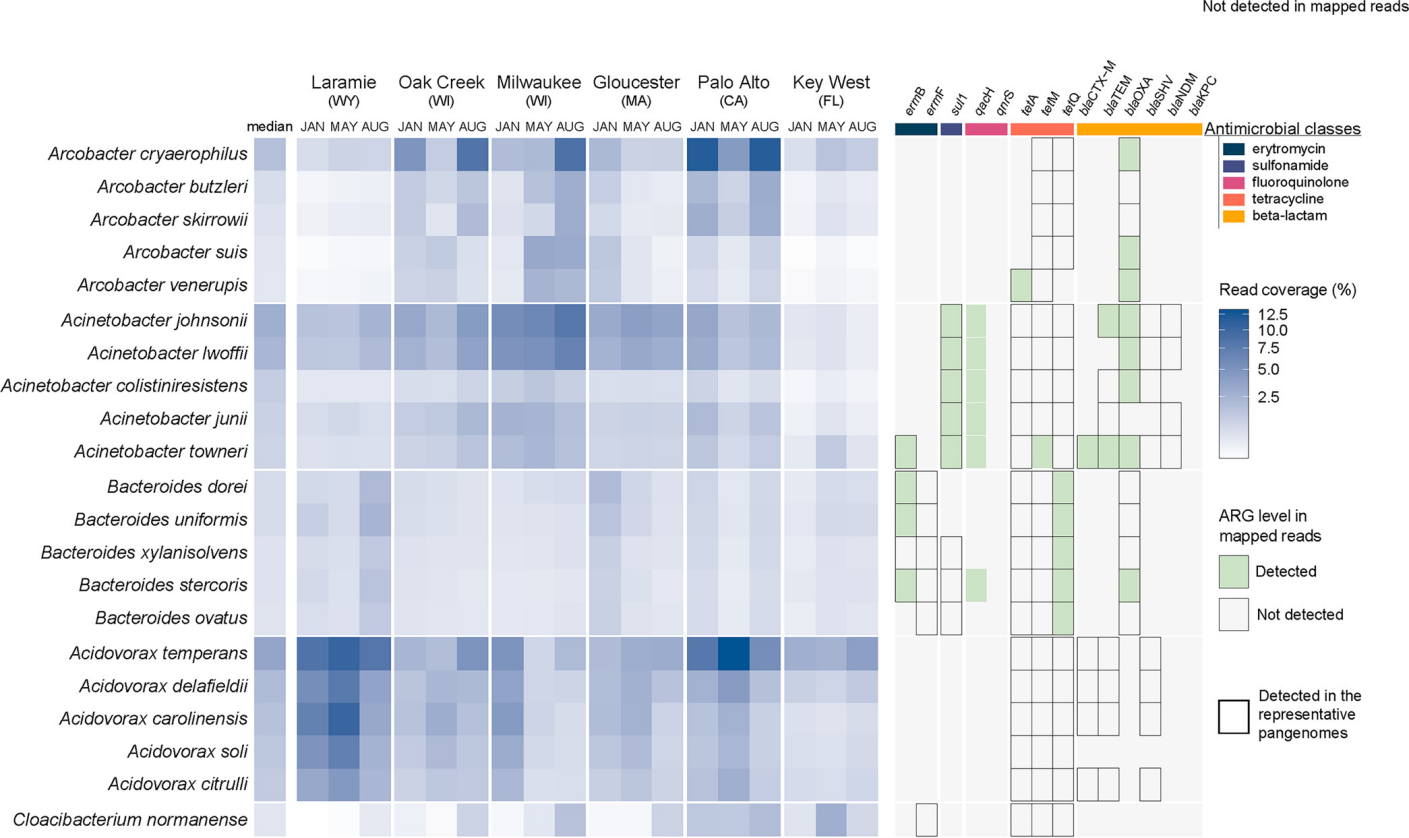
Coverage of published representative pangenomes (row) across the 18 sewage metagenomes (columns) (left), and detection levels of targeted ARGs among the reads mapped to the representative pangenomes (right). Only the five top *Arcobacter*, Acinetobacter, *Bacteroides*, *Acidovorax*, and *Cloacibacterium* representative pangenomes of each species are displayed (based on the median coverage across the 18 metagenomes). The complete coverage analysis of 603 representative pangenomes is shown in Table S1. Right: green squares show the ARGs detected using DeepARG among all metagenomic reads mapped to the representative pangenomes. Black borders display ARGs detected in the representative pangenomes using blastx. *qacS* was not found in the five most common Acinetobacter species but is present in A. baumannii.

Because fragments of the 16S rRNA gene are not discriminant enough to confidently assign sequences to the species level, we used metagenomic sequencing from 18 of the WWTP samples (representatives from cold and warm cities) to identify the species belonging to the top 10 genera identified using 16S rRNA gene data. To do this, we mapped our metagenomes to 603 NCBI reference genome assemblies. Overall, coverage results confirmed the dominance of putative species detected using 16S data (see Table S1 and Fig. 5 for partial results). In fact, among the NCBI *Arcobacter* genome assemblies, A. cryaerophilus (median coverage of 1.79%) and A. butzleri (0.62%) had the largest coverage. Acinetobacter was dominated by *A. johnsonii* (2.85%), A. lwoffii (2.33%), *A. colistiniresistens* (1.18%), and A. junii (1.01%). *Aeromonas* was dominated by *A. temperans* (3.52%), *A. delafieldii* (2.05%), *A. carolinensis* (1.74%), and *A. soli* (1.47%). *Cloacibacterium*, which is represented by one species in the reference genomes, *C. normanense*, had a median coverage of 0.39%. Unlike the results predicted from the 16S rRNA gene assignments, the coverage of Bacteroides graminisolvens (0.13%) was lower than *B. dorei* (0.66%) and B. uniformis (0.64%). It should be taken into consideration that these analyses are limited to the representative genomes deposited in NCBI databases, and unnamed species within each genus were not included in these analyses.

10.1128/msystems.00118-22.8 TABLE S1 Metagenomic mapping coverage of 603 NCBI published genomes to confirm dominant species detected using 16S data. Download Table S1, XLSX file, 0.10 MB. Copyright © 2022 Roguet et al. 2022 Roguet et al. https://creativecommons.org/licenses/by/4.0/ This content is distributed under the terms of the Creative Commons Attribution 4.0 International license.

Most of the dominant ASVs displayed high (HTP)- or low (LTP)-temperature preferences (Fig. 4b). In some cases, all ASVs belonging to a genus exhibited a temperature preference. For example, all ASVs assigned to *Cloacibacterium* displayed HTP, and all ASVs assigned to *Trichococcus* showed LTP. These 16S rRNA gene preferences were supported by the metagenomic read distributions for the warm versus cold sewage cities (Table S1). Other genera, *Flavobacterium* and *Acidovorax*, had ASVs with temperature and no-temperature preferences, and some abundant genera, including *Arcobacter* and Acinetobacter, had both HTP and LTP ASVs. A focus on *Arcobacter* and Acinetobacter revealed a strong bifurcation in their ASV abundance patterns that was explained largely by the ambient air temperature. Among the *Arcobacter* ASVs assigned to the species level, A. butzleri (oligo_06218) and *A. aquimarinus* (oligo_08706) were preferentially observed in high-temperature samples, while A. suis was preferential to low temperatures (oligo_06232 and oligo_08269). Interestingly, A. cryaerophilus displayed both high (oligo_06304)- and low (oligo_06371)-temperature preferences. Regarding the Acinetobacter assemblage, *A. johnsonii* (oligo_08632) did not display strong temperature preferences, unlike *A. towneri* (oligo_03803), for example, which had a high-preferred temperature.

### Human fecal organisms are a small component of sewage.

None of the dominant ASVs were attributed to human feces as a source (Fig. 4b and c). Overall, ASVs shared with human fecal bacteria identified in the HMP represented a small fraction of the sewer microbiome (~13% of total sequence proportion) (Fig. S6). These ASVs were primarily assigned to *Bacteroidetes* and *Firmicutes* and were classified as NTP-LV, demonstrating a substantial homogeneity of the human signature in sewage regardless of the city investigated. Notably, within the family *Enterobacteriaceae*, which contains common fecal pollution indicators, only 5 of the 30 ASVs were also present in the HMP database. However, one of these ASVs was fairly abundant (maximum abundance >5%; Fig. 4c).

10.1128/msystems.00118-22.6 FIG S6 Proportion of the ASVs sharing 100% of similarity with the Human Microbiome Project (HMP) stool V3–V5 database. Values in parenthesis indicate the number of representative ASVs matching the HMP database out of the total number of ASVs assigned to the families. Families with <0.25% occurrence not shown. Download FIG S6, PDF file, 0.2 MB. Copyright © 2022 Roguet et al. 2022 Roguet et al. https://creativecommons.org/licenses/by/4.0/ This content is distributed under the terms of the Creative Commons Attribution 4.0 International license.

The V4–V5 sequences of the gene markers commonly used to track human fecal contamination, i.e., HF183 and Lachno3, were ubiquitously recovered across the nonindustrial sewer systems (oligo_04154 and oligo_01392, respectively). However, their maximum relative abundance never exceeded 2.5%, and their median abundance was 0.3%, suggesting that additional indicators specific to sewer systems could offer a more sensitive measure for surveying the sewage signal in the environment.

### Antibiotic resistance profiles of high-abundance organisms.

A DeepARG analysis was performed on reads recruited to the 603 NCBI genomes of the predominant *Arcobacter*, Acinetobacter, *Bacteroides*, *Acidovorax*, and *Cloacibacterium* species (Fig. 5). This process allowed us to use the metagenomic data to assess the occurrence of specific antibiotic resistance genes (ARGs) associated with these organisms. We found that *bla*OXA was present in both the *Arcobacter* and Acinetobacter reads. Furthermore, *sul1* was enriched in Acinetobacter spp., and *ermB* and *tetQ* were enriched in *Bacteroides* spp. (Fig. 5). Since we only assessed ARG occurrence in the representative genomes from reference species in NCBI, some classes of ARGs previously reported within these genera (particularly *Arcobacter* and Acinetobacter) may not be reflected in this analysis.

## DISCUSSION

### Sewer systems, a unique environment with a consistent bacterial signature.

The wastewater arriving at treatment plants provides an integrative sample of the microbial communities harbored in the vast network of pipes beneath our cities. Similar to how we have gained insight into the microbial ecology of the human gut using fecal samples, wastewater samples can reflect the metabolic potential of the resident community members, in addition to surveying the human-derived microbiome passing through the system. This study entailed a large-scale effort to characterize these microbial populations with a comprehensive U.S. geographic and seasonal representation and includes a range of system sizes and ages. Each of the 71 U.S. and 1 Spain sewer systems investigated had a microbial taxonomic composition consistent with sewer microbiomes reported worldwide (7, 34–36).

The predominance of the same ASVs in gravity sewer systems worldwide underlines that just like other terrestrial and aquatic (e.g., soils, lakes, or oceans) or host-associated (e.g., human gut) systems, sewer systems are a highly defined environment that exert similar and/or convergent selective pressures, which results in bacterial assemblages with ecologically coherent life strategies or functional traits (37). A high prevalence of co-occurring members across the 208 samples used in this study may indicate the presence of metabolic networks that could be explored for either their detrimental effects or beneficial metabolisms. To draw an analogy to the gut microbiome of mammals (38–40), this urban gut microbiome is shaped primarily by the “diet” of the host, through human fecal, urine, and household waste loads, creating a common substrate landscape worldwide. Alterations to typical municipal waste streams seem to have a large impact on the resultant community. For instance, the predominant *Arcobacter* ASV (oligo_06304), detected in all domestic sewer systems, was not detected in the industrial sewer networks, suggesting that high industrial waste discharges and/or low human loads affect the composition of a typical sewer microbiome.

### Temperature-driven sewer ecotypes.

The relative abundance patterns of a large fraction of the nonfecal-derived ASVs exhibited strong high- or low-temperature preferences regardless of the geographic distances between the cities. The result was a consistent association of some ASVs with cities with a warmer climate and other ASVs with cities with a colder climate (Fig. S2). Results from this large scale comparison are congruent with our recent publication exploring the temporal dynamics of the bacterial community over a 5-year monthly time-series within a single city (Milwaukee, WI, USA) (14). That work showed a striking seasonal cycle that was strongly correlated to wastewater temperature. Similar observations were reported in German influent samples (36). The 5-year Milwaukee study at two treatment plants demonstrated that other water quality parameters, including total suspended solids, biological oxygen demand (BOD), system flow, or nutrients had either weaker correlations than wastewater temperature or no correlation to the change in microbial community composition. Of those parameters that were correlated, all were additionally correlated with wastewater temperature.

In the present study, we found flow or population size did not explain the bacterial community variability, whereas temperature explained the most variability (Fig. S3) concurrent with the recovery of strong temperature patterns (Fig. 3). We did not further investigate a global relationship of wastewater parameters to community composition since each city only had three samples and individual system parameters are often dependent on each other (i.e., amount of gravity flow impacting BOD). Wastewater temperature was not available for many of the plants in our study, or was not measured in a uniform manner, therefore, we used the past 30-year average daily low air temperatures on the collection date as a proxy for the climate of that city during the sampling time. We note that recent studies in the Milwaukee system show the temperature range measured in sensors within the system is narrower and time-lagged compared to air temperature (14, 41). While this makes it difficult to relate an actual temperature to the warm or cold ASVs ecotypes, this method did allow us to bin cities by their general climate at the time of sampling, which provides a useful metric that may be more accessible than wastewater temperature for extrapolating to other urban sewer systems.

Most of the predominant bacterial genera in sewers exhibited temperature preferences. The *Cloacibacterium* genus showed high-temperature preferences, while overall, *Acidovorax*, *Flavobacterium*, *and Trichococcus* genera presented opposite temperature preferences, confirming that previous observations from a single city (14, 18) were globally applicable. Other genera, including the most dominant organisms Acinetobacter and *Arcobacter*, presented contrasted temperature preferences among their members. A preliminary study with the samples from 12 of the 71 U.S. cities and the samples from Reus, Spain showed the two most dominant *Arcobacter* ASVs were at similar levels at a averaged monthly air temperature below 20°C but diverged greatly above this temperature, with one increasing and the other decreasing in relative abundance (17). Warm temperatures appeared to favor ASVs assigned to A. butzleri and *A. aquimarinus*, while cold waters favored A. suis (42). In contrast, A. cryaerophilus members presented both warm and cold temperature preferences. This differentiation may be explained by the lack of taxonomic classification of short 16S rRNA gene reads beyond the species level (43) or the existence of four genomovar clusters within this species having contrasted ecological niches (44). The importance of temperature for modulating microbial communities is a common feature of aquatic habitats (45, 46). In other ecosystems, colder temperatures may favor aerobic conditions (12); modify biotic interactions, including competition, grazing, or viral lysis (47); or alter competitive balances through growth rate modulation. It would be of interest in future work to determine if the within-species ecotypes have unique metabolic activities or alternatively, have redundant capabilities but are adapted to different temperature ranges.

### Ecology of the most abundant bacterial groups in sewers.

Most of the predominant pipe residents, including *Arcobacter*, *Acidovorax*, *Aeromonas*, *Cloacibacterium*, and *Trichococcus* members have been described as capable of facultative anaerobic (30, 48–51) or aerotolerant anaerobic growth (52). This suggests that oxygen tolerance/use flexibility is a key factor in surviving the oxic-anoxic fluctuations that are common to gravity sewer systems.

*Arcobacter* and Acinetobacter have been highlighted as part of the core genera in sewage worldwide (13). They even have been found in Hong Kong (China) sewer systems, where toilets are flushed with seawater (19, 53), even though salinity is a major environmental determinant of microbial community composition (54). In this study, we show that at a very fine taxonomic level, i.e., ASVs, there are abundant core members of these genera in nearly all (>95%) of the 71 U.S. cities examined.

In wastewater, both *Arcobacter* and Acinetobacter members have been described as chemoautotrophic nitrate-reducing, sulfide-oxidizing bacteria under anaerobic conditions (55–57), showing their ability to oxidize sulfide to sulfur or sulfate or using nitrate and/or nitrite as the electron acceptor through the dissimilarity and assimilatory nitrate reduction to ammonia pathways, respectively (58–60). Thus, similar to other sulfur-oxidizing bacteria, *Arcobacter* and Acinetobacter may have a selective advantage in sewage environments by tolerating higher concentrations of hydrogen sulfide and growing at very low-molecular-oxygen concentrations (61). In addition, some *Arcobacter* species, including A. cryaerophilus strains isolated from wastewaters, were observed/predicted to produce hydrogen sulfide (62–64), which can contribute to pipe corrosion in sewer systems, a highly detrimental effect of bacterial metabolism within these systems.

### Human bacterial signatures in sewage.

Understanding the composition, function, and ecology of these systems is not only important for treating waste, but for monitoring the health of human populations, as the COVID-19 pandemic has illustrated. Overall, the human fecal signature within the sewer microbiome was highly consistent and homogeneous across the cities. Our team previously reported that this signature reflects the fecal microbial community of human populations, allowing for the capture and prediction of global health traits, e.g., obesity (20). Other applications include tracking antibiotic resistant bacteria and biomarkers of human health (65–68).

In this study, about 30% of the ASVs belonged to fecal-associated families, but only half (13% of the community) matched the human fecal microbiome. Congruent with the proportion observed in other studies (14, 15), this result underlines that specific taxa within commonly fecal-associated families (e.g., *Bacteroidaceae*, *Lachnospiraceae*, *Ruminococcaceae*) are likely pipe-associated members. In fact, pipe-associated ASVs belonging to fecal-associated families displayed strong temperature-driven preferences and were not recovered within the same network as the fecal bacteria (Fig. S7). Previous work by Feng and McLellan (69) demonstrated that the most abundant *Bacteroides* was not associated with human stool. Interestingly, in this study, *Enterobacteriaceae* had ASVs that matched the human fecal microbiome but also presented temperature preferences. Although these findings may reflect actual ecological preferences of gut-specific taxa in a secondary habitat (70), they may also be a result of false-positive matches between sewage ASVs and the Human Microbiome Project databases due to the low resolution of short 16S rRNA gene amplicons. Nevertheless, it is interesting to note that *Enterobacteriaceae* comprise a small fraction of the stool microbiome of healthy individuals (40, 70) but represented ~2% of the sewer microbiome overall. This enrichment may be of concern since *Enterobacteriaceae* produces extended-spectrum beta-lactamases, including carbapenemases that have emerged over the last two decades as a major antimicrobial-resistance health concern (71).

Human-specific markers, e.g., human *Bacteroides* or human *Lachnospiraceae* (72, 73), have been developed to track human fecal pollution in the environment. However, these markers do not represent a large fraction of the overall sewer microbiome. Using markers specifically associated with sewer infrastructure could increase sensitivity for monitoring human sewage contamination in the environment (13). *Arcobacter* and Acinetobacter are in extremely high abundance in untreated sewage, and while both have been isolated independently from human and animal hosts and other habitats such as agricultural soils and polluted industrial environments (74, 75), their co-occurrence is indicative of sewage contamination (76–79). Tracking a suite of these organisms could serve as a microbial signature for contamination from sewer infrastructure (76, 80).

### Antibiotic resistance gene reservoirs in dominant organisms.

There are ongoing concerns that sewer systems are hot spots for ARGs in sewers (7, 81, 82). However, within sewer systems, many ARGs are likely primarily associated with pipe-resident rather than human-associated bacteria. This is an important distinction if untreated wastewater is going to be used to survey for ARGs (23, 83). The two dominant genera *Arcobacter* and Acinetobacter contain multiclass ARGs (24, 27, 84), and specific species within these genera are considered opportunistic pathogens. Some ARGs may be intrinsic to the organisms and present in ancestorial strains (85). Furthermore, the presence of multiple ARGs might underline the strong biotic pressures in sewers (e.g., competition) and/or the constant trace levels of chemicals, which may favor the enrichment of ARGs as ultimate survival strategies (86). We identified multiple extended spectrum beta-lactamase (ESBL) ARGs in the metagenomic data mapped to the pangenomes of *Arcobacter*, Acinetobacter, and other highly abundant members, but also did not identify some previously reported ARGs associated with these genera. We utilized multiple genomes (up to 50) obtained from NCBI to represent the species of the dominant organisms. Mapping of metagenomic reads recovered patterns similar to ASV distribution across these samples; however, the density of metagenomic reads may not be accurate since reads may be recruited to highly conserved regions of the 16S rRNA gene or other conserved genomic features such as transposons or phages that would result in a false positive signal. Our analysis was limited by the number and sources of published *Arcobacter* and Acinetobacter genomes, highlighting the need for cultured representatives and a more detailed analysis of the genomic content of these dominant genera from the sewer system environment.

The human-derived component of the sewage microbial community is most relevant for wastewater surveillance of ARGs. The potential propagation of *Enterobacteriaceae* and the large reservoir of ESBL genes within highly abundant resident pipe bacteria confound these assessments and require a more comprehensive understanding of their ecology before wastewater surveillance for these targets would be interpretable.

### Conclusions.

Sewer infrastructures are new habitats for unique microbiomes and are increasingly being recognized as a means to monitor human populations. With the high biomass of resident organisms and high metabolic potential of sewer microbiota, identifying functional guilds could lead to the engineering of this community to reduce harmful activity and enhance beneficial functions. In this study, we conducted a large-scale survey of 71 cities over three distinct seasons that allowed us to identify ubiquitous sewage members and common distribution patterns. The composition of the sewer microbiome exhibited a surprising consistency at a high resolution, i.e., ASV level, across all systems explored, suggesting characterizations of these systems could be extrapolated globally. Furthermore, the COVID-19 pandemic has illustrated that sewer systems can act as an integrated sample of large human populations (67, 87, 88). As we expand efforts past the current pandemic crisis, monitoring for clinically relevant antibiotic resistance traits and the organisms that contain them will become more important (68). A more comprehensive understanding of the organisms that serve as reservoirs will be critical in this effort. Sewer infrastructure represents the guts of a city, and in a sense, it resembles the gastrointestinal tract of an individual. The way the gastrointestinal tract is studied extensively to understand the health and the quality of life of a single individual, much can be learned about the same metrics for a given city and its inhabitants by keeping an eye on the sewer infrastructure.

## MATERIALS AND METHODS

### Sample collection.

Sewage influent samples from 71 cities and 78 wastewater treatment plants (WWTPs) across the United States and one city in Spain were collected during August 2012, January 2013, and May 2013 as part of former surveys. Details on the collection method can be found in (17, 20). Briefly, 1 liter of single-time point grab or 24-h flow-weighted composite samples were collected and shipped overnight on ice. A total of 25-mL subsamples were filtered through 0.22-μm mixed cellulose ester filters (47-mm diameter; Millipore, Billerica, MA, USA) and stored in 2-mL screw-cap freezer tubes at −80°C. The list of the samples and their associated metadata are reported in Data Set S1.

### DNA extraction.

DNA from filters was extracted using the FastDNA spin kit for soil (MP Biomedicals, Solon, OH, USA) according to the manufacturer’s instructions. One modification of this protocol was applied: Cells were mechanically lysed using a MiniBeadBeater-8 cell disruptor (BioSpec Products, Bartlesville, OK, USA) for 1 min at room temperature. Crude DNA extracted was purified using the PowerCleanDNA cleanup kit (MoBio Laboratories, Carlsbad, CA, USA), and stored at −20°C until further processing.

### 16S rRNA gene sequencing and library construction.

Amplicon libraries were constructed at the Josephine Bay Paul Center at the Marine Biological Laboratory (Woods Hole, MA, USA) using the MiSeq Illumina platform. Details for amplicon library construction and sequencing procedures for the V4 to V5 regions are described in Morrison et al. (89). Trimming, quality-filtering and merging procedures are described in Newton et al. (20). Raw sequences are archived in the National Center for Biotechnology Information Sequence Read Archive under BioProjects PRJNA261344 and PRJNA264400. Processed sequenced can be retrieved from the website Visualization and Analysis of Microbial Population Structures; https://vamps2.mbl.edu/) under the project name SLM_CITY_Bv4v5 (90).

We rarefied each sample to 80,000 sequences. Rarefied samples were analyzed by minimum entropy decomposition, an unsupervised sensitive oligotyping method that uses Shannon entropy to partition amplicon sequence data sets into homogeneous units, so-called ASVs, that can differ from each other by as few as a single base pair (91). Performed using the oligotyping pipeline version 2.1, this analysis distinguishes DNA sequence differences in nucleotides originating from true genetic variation among organisms from noise due to sequencing errors. ASVs that do not meet the minimum substantive abundance (M) criterion were discarded. M was set to 300. Sequences were taxonomically assigned using RDP classifier (92) with an 80% bootstrap value.

### Shotgun sequencing, library construction, and processing.

We sequenced the sewage metagenome for six U.S. cities in all three sample periods. Shotgun metagenomic libraries were prepared with OVATION Ultralow protocol (NuGen) and used an Illumina NextSeq 500 platform to generate 2 × 150 nt paired-end sequencing reads. Low-quality reads were removed using the ‘iu-filter- quality-minoche’ command in illumina-utils v1.4.1 (93). We did not succeed in assembling high-quality genomes from the sewage metagenomes using MEGAHIT (94) using the workflow described by Delmont et al. (95), likely due to the high bacterial diversity and diverse populations with a genus or species in this complex matrix. Raw sequences were deposited to the NCBI Sequence Read Archive under the BioProject PRJNA801794.

The coverage of representative species belonging to predominant genera in sewage was evaluated across the 18 sewage metagenomes. We screened a total of 3,794 genome assemblies deposited on NCBI GenBank and RefSeq databases. A maximum of 50 genomes were used to represent a species. We removed 1,755 redundant genomes with the function ‘anvi-dereplicate-genomes’ in anvi’o v6.1 (96) using the program PyANI (97) with the default parameters and a similarity threshold of 0.99. Dereplicated pangenomes for 603 species were concatenated and were used to recruit reads from metagenomes using the default parameters of Bowtie2 (98). The list of the NCBI deposited genomes used in this analysis is detailed in Table S2. Finally, we performed a DeepARG analysis (99) using the online platform (using 60% identity and 50% coverage) to explore the presence of antibiotic resistance genes in the recruited reads of the five predominant species of the five top genera. For each species, the BAM files listing recruited reads across the 18 metagenomes were concatenated and converted into fastq using SAMtools v1.10 (100) prior to the analysis. In parallel, we also explored the presence of these genes in the representative pangenomes with the command line blastx (101) (e-value <1e-50) using the DeepARG database accessible through the command ‘deeparg download_data.’

10.1128/msystems.00118-22.9 TABLE S2 List of NCBI assemblies used to construct representative pangenomes. Download Table S2, XLSX file, 0.1 MB. Copyright © 2022 Roguet et al. 2022 Roguet et al. https://creativecommons.org/licenses/by/4.0/ This content is distributed under the terms of the Creative Commons Attribution 4.0 International license.

### Human Microbiome Project comparison.

We used the Human Microbiome Project (HMP) to identify among our ASVs, which shared 100% identity with the human stool sequences. We used the files generated by QIIME on the HMPv35 data set, including the v1.3.0-dev OTU table (https://www.hmpdacc.org/hmp/HMQCP/). This data set was obtained by targeting the region V3 to V5 of the 16S rRNA gene. Only stool samples not listed as mislabeled or contaminated and with a sequencing depth higher than 2,000 sequences (corresponding to the lower quartile of sequences in the stool samples) were considered. Singletons were discarded. A total of 223 stool samples composed of 9,650 OTUs were used to compare our ASVs to the HMPv35 data sets. An ASV was considered “detected in the stool HMPv35 data set” when its exact sequence was found in the reverse complement of at least one of the 9,650 OTUs composing the stool HMPv35 data set.

### Earth Microbiome Project analysis.

We used the Earth Microbiome Project (EMP) data set to explore bacterial community structure differences between our 16S sewage samples and other aquatic and terrestrial habitats. Data were gathered from the EMP database “emp_cr_silva_16S_123.subset_2k.biom” (http://ftp.microbio.me/emp/release1/). To limit the bacterial dissimilarities associated with dispersal limitation, we only considered samples collected in the United States and labeled as air, freshwater, freshwater sediment, human gut, indoor, marine, marine sediment, mine drainage, and soil metagenomes. Because the EMP primers target the region V4 of the 16S rRNA gene we trimmed our data set to the EMP primer positions (102) using Cutadapt (103).

### Environmental parameters.

Chemical and physical measurements were provided by the WWTPs (see Data Set S1 for details). Moreover, we aggregated data for the past 30-year average daily low air temperatures on the collection date for each sample from National Oceanic and Atmospheric Administration (NOAA) National Climatic Data Center (http://www.ncdc.noaa.gov) On the day of the sampling collection, 1-meter-deep soil temperatures were also extracted from the NOAA database, when meteorological stations were located within 100 km of the WWTPs (*n* = 30). Other parameters were estimated, including the size, the median age, and the percentage of obese in the served population. Refer to Newton et al. (20) for calculation details.

### Network analysis.

Co-occurrence networks were constructed for each sampling campaign based on counts (zero left blank) of the 250 top ASVs using Molecular Ecological Network Analyses pipeline (http://ieg4.rccc.ou.edu/mena/login.cgi) (104). Pairwise similarity was calculated for each ASV pair using Pearson correlation coefficients. Random matrix theory-based method (using Gaussian orthogonal ensemble statistics and Poisson distribution) was used to automatically identify the appropriate similarity threshold (*P* < 0.05) to construct the networks. Network modularity was characterized using the greedy modularity optimization method (105). Furthermore, within-module connectivity (Z_i_) and among-module connectivity (P_i_) of each node were calculated to classify nodes into four categories: module hubs (Z_i_ > 2.5 and P_i_ < 0.6), network hubs (Z_i_ > 2.5 and P_i_ > 0.6), connectors (Z_i_ < 2.5 and P_i_ > 0.6), and peripherals (Z_i_ < 2.5 and P_i_ < 0.6) (106). Networks were visualized using Cytoscape 3.7.2 (107).

### Statistical analysis.

All statistical analyses were performed using R v.3.6.3 (108). The relative importance of spatial (latitude, longitude), environmental (air temperature), population (size, age, and obesity), and WWTP attributes (grab/composite sample, separated/combined systems, and average daily flow) factors was assessed by decomposing the bacterial community variation (109) using the function varpart in the R package *vegan* (110). Only the 250 most abundant ASVs were considered in this analysis. Qualitative variables were transformed into dummy variables. Qualitative variables were centered and scaled. Redundancy analysis (RDA) was performed on the sets of factors that explained the most variance of the assemblages, i.e., spatial parameters (6%) and temperature (9%), to identify which variable better explained the shift in the community. We did not include physical-chemical parameters (e.g., total phosphate, oxygen) in this analysis due to the limited data collected across the 211 samples. However, RDA performed on a subset of samples indicated that their importance was minor compared to the spatial parameters or the temperature (data not shown). We identified at which temperature the bacterial community assemblage was shifting using random forest regression (constructed with 200 trees) implemented in the package *randomForest* (111). We used the function *plot.getTree* in the package *reprtree* (112) to draw the decision tree generated by random forest. We set the temperature breakpoint as the value associated with the first decision tree split. The breakpoint was estimated using atmospheric temperature and not ground temperature, which is more likely to reflect temperature in wastewater systems. The Correlation between air and soil temperatures was 0.83 (Pearson correlation, *n* = 30, *P* < 0.001). A linear model was performed using the function *lm* on these two variables. The normality of variables and the distribution of residuals were verified. The function *predict* was used to estimate the soil temperature at a 95% level of confidence from air temperature. Nonmetric multidimensional scaling (NMDS) analysis (k = 2) was performed using the R package *vegan* to compare the bacterial community composition between sewage and the distinct terrestrial/aquatic microbiomes (see Earth Microbiome Project microbiomes listed above). Figure 3a was realized using anvi’o (96). We used an NMDS analysis (k = 2) (Fig. S7) to determine the temperature preferences of the ASVs not classified to any of the four temperature preferences using correlations (Figure 3b).

## References

[1] Huang D, Liu X, Jiang S, Wang H, Wang J, Zhang Y. 2018. Current state and future perspectives of sewer networks in urban China. Front Environ Sci Eng 12:2. doi:10.1007/s11783-018-1023-1.

[2] Singh S, Singh S, Kumar V, Kumar S, Singh Dhanjal D, Romero R, Datta S, Bhadrecha P, Singh J. 2020. Microbial remediation for wastewater treatment, p 57–72. *In* Arora PK (ed), Microbial technology for health and environment. Springer, Singapore.

[3] Alvarino T, Suarez S, Lema J, Omil F. 2018. Understanding the sorption and biotransformation of organic micropollutants in innovative biological wastewater treatment technologies. Sci Total Environ 615:297–306. doi:10.1016/j.scitotenv.2017.09.278. 28982079

[4] Wagner M, Loy A. 2002. Bacterial community composition and function in sewage treatment systems. Curr Opin Biotechnol 13:218–227. doi:10.1016/S0958-1669(02)00315-4. 12180096

[5] Rodenburg LA, Du S, Fennell DE, Cavallo GJ. 2010. Evidence for widespread dechlorination of polychlorinated biphenyls in groundwater, landfills, and wastewater collection systems. Environ Sci Technol 44:7534–7540. doi:10.1021/es1019564. 20828204

[6] Liu Y, Dong Q, Shi H. 2015. Distribution and population structure characteristics of microorganisms in urban sewage system. Appl Microbiol Biotechnol 99:7723–7734. doi:10.1007/s00253-015-6661-7. 25981998

[7] Auguet O, Pijuan M, Borrego CM, Rodriguez-Mozaz S, Triadó-Margarit X, Giustina SV, Gutierrez O. 2017. Sewers as potential reservoirs of antibiotic resistance. Sci Total Environ 605–606:1047–1054. doi:10.1016/j.scitotenv.2017.06.153. 28709370

[8] Cayford BI, Dennis PG, Keller J, Tyson GW, Bond PL. 2012. High-throughput amplicon sequencing reveals distinct communities within a corroding concrete sewer system. Appl Environ Microbiol 78:7160–7162. doi:10.1128/AEM.01582-12. 22843532 PMC3457466

[9] Li W, Zheng T, Ma Y, Liu J. 2019. Current status and future prospects of sewer biofilms: Their structure, influencing factors, and substance transformations. Sci Total Environ 695:133815. doi:10.1016/j.scitotenv.2019.133815. 31416035

[10] Shi X, Ngo HH, Sang L, Jin P, Wang XC, Wang G. 2018. Functional evaluation of pollutant transformation in sediment from combined sewer system. Environ Pollut 238:85–93. doi:10.1016/j.envpol.2018.03.007. 29547865

[11] Xing Y, Chen X, Wang S, Zhang Z, Liu X, Lu J. 2021. Effect of minocycline on the changes in the sewage chemical index and microbial communities in sewage pipes. J Hazard Mater 402:123792. doi:10.1016/j.jhazmat.2020.123792. 33254801

[12] Gudjonsson G, Vollertsen J, Hvitved-Jacobsen T. 2002. Dissolved oxygen in gravity sewers - Measurement and simulation. Water Sci Technol 45:35–44. doi:10.2166/wst.2002.0049. 11902480

[13] McLellan SL, Roguet A. 2019. The unexpected habitat in sewer pipes for the propagation of microbial communities and their imprint on urban waters. Curr Opin Biotechnol 57:34–41. doi:10.1016/j.copbio.2018.12.010. 30682717 PMC7018504

[14] LaMartina E, Mohaimani AA, Newton RJ. 2021. Urban wastewater bacterial communities assemble into seasonal steady states. Microbiome 9:1. doi:10.1186/s40168-020-00939-1. 34016155 PMC8139061

[15] McLellan SL, Huse SM, Mueller-Spitz SR, Andreishcheva EN, Sogin ML. 2010. Diversity and population structure of sewage-derived microorganisms in wastewater treatment plant influent. Environ Microbiol 12:378–392. doi:10.1111/j.1462-2920.2009.02075.x. 19840106 PMC2868101

[16] Buelow E, Bayjanov JR, Majoor E, Willems RJL, Bonten MJM, Schmitt H, van Schaik W. 2018. Limited influence of hospital wastewater on the microbiome and resistome of wastewater in a community sewerage system. FEMS Microbiol Ecol 94:fiy087. doi:10.1093/femsec/fiy087. 29767712

[17] Fisher JC, Levican A, Figueras MJ, McLellan SL. 2014. Population dynamics and ecology of Arcobacter in sewage. Front Microbiol 5:1. doi:10.3389/fmicb.2014.00001. 25426103 PMC4224126

[18] Vandewalle JL, Goetz GW, Huse SM, Morrison HG, Sogin ML, Hoffmann RG, Yan K, McLellan SL. 2012. Acinetobacter, Aeromonas and Trichococcus populations dominate the microbial community within urban sewer infrastructure. Environ Microbiol 14:2538–2552. doi:10.1111/j.1462-2920.2012.02757.x. 22524675 PMC3427404

[19] Cai L, Ju F, Zhang T. 2014. Tracking human sewage microbiome in a municipal wastewater treatment plant. Appl Microbiol Biotechnol 98:3317–3326. doi:10.1007/s00253-013-5402-z. 24305737

[20] Newton RJ, McLellan SL, Dila DK, Vineis JH, Morrison HG, Murat Eren A, Sogin ML. 2015. Sewage reflects the microbiomes of human populations. mBio 6:e02574-14. doi:10.1128/mBio.02574-14. 25714718 PMC4358014

[21] Ibarbalz FM, Orellana E, Figuerola ELM, Erijman L. 2016. Shotgun metagenomic profiles have a high capacity to discriminate samples of activated sludge according to wastewater type. Appl Environ Microbiol 82:5186–5196. doi:10.1128/AEM.00916-16. 27316957 PMC4988215

[22] Dottorini G, Michaelsen TY, Kucheryavskiy S, Andersen KS, Kristensen JM, Peces M, Wagner DS, Nierychlo M, Nielsen PH. 2021. Mass-immigration determines the assembly of activated sludge microbial communities. Proc Natl Acad Sci USA 118:e2021589118. doi:10.1073/pnas.2021589118. 34187887 PMC8271747

[23] Zhang AN, Gaston JM, Dai CL, Zhao S, Poyet M, Groussin M, Yin X, Li LG, van Loosdrecht MCM, Topp E, Gillings MR, Hanage WP, Tiedje JM, Moniz K, Alm EJ, Zhang T. 2021. An omics-based framework for assessing the health risk of antimicrobial resistance genes. Nat Commun 12:4765. doi:10.1038/s41467-021-25096-3. 34362925 PMC8346589

[24] Millar JA, Raghavan R. 2017. Accumulation and expression of multiple antibiotic resistance genes in *Arcobacter cryaerophilus* that thrives in sewage. PeerJ 5:e3269. doi:10.7717/peerj.3269. 28462059 PMC5407278

[25] Zhang Y, Marrs CF, Simon C, Xi C. 2009. Wastewater treatment contributes to selective increase of antibiotic resistance among Acinetobacter spp. Sci Total Environ 407:3702–3706. doi:10.1016/j.scitotenv.2009.02.013. 19321192

[26] Harnisz M, Korzeniewska E. 2018. The prevalence of multidrug-resistant Aeromonas spp. in the municipal wastewater system and their dissemination in the environment. Sci Total Environ 626:377–383. doi:10.1016/j.scitotenv.2018.01.100. 29353783

[27] Hultman J, Tamminen M, Pärnänen K, Cairns J, Karkman A, Virta M. 2018. Host range of antibiotic resistance genes in wastewater treatment plant influent and effluent. FEMS Microbiol Ecol 94:fiy038. doi:10.1093/femsec/fiy038. PMC5939699 29514229

[28] Baas-Becking LGM. 1934. Geobiologie of inleiding tot de milieukunde. WP Van Stockum & Zoon NV, The Hague, The Netherlands.

[29] Jensen H, Biggs CA, Karunakaran E. 2016. The importance of sewer biofilms. Wiley Interdiscip Rev Water 3:487–494. doi:10.1002/wat2.1144.

[30] Allen TD, Lawson PA, Collins MD, Falsen E, Tanner RS. 2006. Cloacibacterium normanense gen. nov., sp. nov., a novel bacterium in the family Flavobacteriaceae isolated from municipal wastewater. Int J Syst Evol Microbiol 56:1311–1316. doi:10.1099/ijs.0.64218-0. 16738108

[31] Monfort P, Baleux B. 1990. Dynamics of Aeromonas hydrophila, Aeromonas sobria, and Aeromonas caviae in a sewage treatment pond. Appl Environ Microbiol 56:1999–2006. doi:10.1128/aem.56.7.1999-2006.1990. 2389929 PMC184551

[32] Malik A, Sakamoto M, Ono T, Kakii K. 2003. Coaggregation between Acinetobacter johnsonii S35 and Microbacterium esteraromaticum strains isolated from sewage activated sludge. J Biosci Bioeng 96:10–15. doi:10.1016/S1389-1723(03)90090-9. 16233476

[33] Nishiyama T, Ueki A, Kaku N, Watanabe K, Ueki K. 2009. Bacteroides graminisolvens sp. nov., a xylanolytic anaerobe isolated from a methanogenic reactor treating cattle waste 1901–1907. Int J Syst Evol Microbiol 59:1901–1907. doi:10.1099/ijs.0.008268-0. 19567576

[34] Lee SH, Kang HJ, Park HD. 2015. Influence of influent wastewater communities on temporal variation of activated sludge communities. Water Res 73:132–144. doi:10.1016/j.watres.2015.01.014. 25655320

[35] Greay TL, Gofton AW, Zahedi A, Paparini A, Linge KL, Joll CA, Ryan UM. 2019. Evaluation of 16S next-generation sequencing of hypervariable region 4 in wastewater samples: An unsuitable approach for bacterial enteric pathogen identification. Sci Total Environ 670:1111–1124. doi:10.1016/j.scitotenv.2019.03.278. 31018427

[36] Numberger D, Ganzert L, Zoccarato L, Mühldorfer K, Sauer S, Grossart HP, Greenwood AD. 2019. Characterization of bacterial communities in wastewater with enhanced taxonomic resolution by full-length 16S rRNA sequencing. Sci Rep 9:1. doi:10.1038/s41598-019-46015-z. 31273307 PMC6609626

[37] Philippot L, Andersson SGE, Battin TJ, Prosser JI, Schimel JP, Whitman WB, Hallin S. 2010. The ecological coherence of high bacterial taxonomic ranks. Nat Rev Microbiol 8:523–529. doi:10.1038/nrmicro2367. 20531276

[38] David LA, Maurice CF, Carmody RN, Gootenberg DB, Button JE, Wolfe BE, Ling AV, Devlin AS, Varma Y, Fischbach MA, Biddinger SB, Dutton RJ, Turnbaugh PJ. 2014. Diet rapidly and reproducibly alters the human gut microbiome. Nature 505:559–563. doi:10.1038/nature12820. 24336217 PMC3957428

[39] Nishida AH, Ochman H. 2018. Rates of gut microbiome divergence in mammals. Mol Ecol 27:1884–1897. doi:10.1111/mec.14473. 29290090 PMC5935551

[40] Ley RE, Hamady M, Lozupone C, Turnbaugh PJ, Roy R, Bircher JS, Schlegel ML, Tucker TA, Mark D, Knight R, Gordon JI, Ramey RR, Bircher JS, Schlegel ML, Tucker TA, Schrenzel MD, Knight R, Gordon JI. 2008. Evolution of mammals and their gut microbes. Science 320:1647–1651. doi:10.1126/science.1155725. 18497261 PMC2649005

[41] Schussman MK, Mclellan SL. 2022. Effect of time and temperature on SARS-CoV-2 in municipal wastewater conveyance systems. Water 14:1373. doi:10.3390/w14091373.

[42] Levican A, Collado L, Yustes C, Aguilar C, Figueras MJ. 2014. Higher water temperature and incubation under aerobic and microaerobic conditions increase the recovery and diversity of arcobacter spp. from shellfish. Appl Environ Microbiol 80:385–391. doi:10.1128/AEM.03014-13. 24185851 PMC3911005

[43] Jeong J, Yun K, Mun S, Chung WH, Choi SY, Nam Y, Lim MY, Hong CP, Park CH, Ahn Y, Han K. 2021. The effect of taxonomic classification by full-length 16S rRNA sequencing with a synthetic long-read technology. Sci Rep 11:1–12. doi:10.1038/s41598-020-79139-8. 33462291 PMC7814050

[44] Pérez-Cataluña A, Collado L, Salgado O, Lefiñanco V, Figueras MJ. 2018. A polyphasic and taxogenomic evaluation uncovers Arcobacter cryaerophilus as a species complex that embraces four genomovars. Front Microbiol 9:805. doi:10.3389/fmicb.2018.00805. 29755434 PMC5934430

[45] Adams HE, Crump BC, Kling GW. 2010. Temperature controls on aquatic bacterial production and community dynamics in arctic lakes and streams. Environ Microbiol 12:1319–1333. doi:10.1111/j.1462-2920.2010.02176.x. 20192972

[46] Ward CS, Yung CM, Davis KM, Blinebry SK, Williams TC, Johnson ZI, Hunt DE. 2017. Annual community patterns are driven by seasonal switching between closely related marine bacteria. ISME J 11:1412–1422. doi:10.1038/ismej.2017.4. 28234350 PMC5437356

[47] Korajkic A, Wanjugi P, Brooks L, Cao Y, Harwood VJ. 2019. Persistence and decay of fecal microbiota in aquatic habitats. Microbiol Mol Biol Rev 83:e00005-19. doi:10.1128/MMBR.00005-19. 31578217 PMC7405076

[48] Fernando DM, Khan IUH, Patidar R, Lapen DR, Talbot G, Topp E, Kumar A. 2016. Isolation and characterization of acinetobacter baumannii recovered from campylobacter selective medium. Front Microbiol 7:1871. doi:10.3389/fmicb.2016.01871. 27917170 PMC5114274

[49] Colwell RR, MacDonell MT, De Ley J. 1986. Proposal to recognize the family Aeromonadaceae fam. nov. Int J Syst Bacteriol 36:473–477. doi:10.1099/00207713-36-3-473.

[50] Vandamme P, De Ley J. 1991. Proposal for a new family, Campylobacteraceae. Int J Syst Bacteriol 41:451–455. doi:10.1099/00207713-41-3-451.

[51] Männistö MK, Puhakka JA. 2002. Psychrotolerant and microaerophilic bacteria in boreal groundwater. FEMS Microbiol Ecol 41:9–16. doi:10.1111/j.1574-6941.2002.tb00961.x. 19709234

[52] Pikuta EV, Hoover RB. 2014. The genus Trichococcus, p 135–145. *In* Holzapfel WH, Wood BJB (ed), Lactic acid bacteria: biodiversity and taxonomy (1st ed.). John Wiley & Sons, Oxford, UK.

[53] Ye L, Zhang T. 2013. Bacterial communities in different sections of a municipal wastewater treatment plant revealed by 16S rDNA 454 pyrosequencing. Appl Microbiol Biotechnol 97:2681–2690. doi:10.1007/s00253-012-4082-4. 22555912 PMC3586070

[54] Lozupone CA, Knight R. 2007. Global patterns in bacterial diversity. Proc Natl Acad Sci USA 104:11436–11440. doi:10.1073/pnas.0611525104. 17592124 PMC2040916

[55] Garcia-de-Lomas J, Corzo A, Carmen Portillo M, Gonzalez JM, Andrades JA, Saiz-Jimenez C, Garcia-Robledo E. 2007. Nitrate stimulation of indigenous nitrate-reducing, sulfide-oxidising bacterial community in wastewater anaerobic biofilms. Water Res 41:3121–3131. doi:10.1016/j.watres.2007.04.004. 17524444

[56] De Gusseme B, De Schryver P, De Cooman M, Verbeken K, Boeckx P, Verstraete W, Boon N. 2009. Nitrate-reducing, sulfide-oxidizing bacteria as microbial oxidants for rapid biological sulfide removal. FEMS Microbiol Ecol 67:151–161. doi:10.1111/j.1574-6941.2008.00598.x. 19120464

[57] Haosagul S, Prommeenate P, Hobbs G, Pisutpaisal N. 2020. Sulfide-oxidizing bacteria community in full scale bioscrubber treating H2S in biogas from swine anaerobic digester. Renew Energy 150:973–980. doi:10.1016/j.renene.2019.11.139.

[58] Miller WG, Parker CT, Rubenfield M, Mendz GL, Wösten MMSM, Ussery DW, Stolz JF, Binnewies TT, Hallin PF, Wang G, Malek JA, Rogosin A, Stanker LH, Mandrell RE. 2007. The complete genome sequence and analysis of the epsilonproteobacterium *Arcobacter butzleri*. PLoS One 2:e1358. doi:10.1371/journal.pone.0001358. 18159241 PMC2147049

[59] Waite DW, Vanwonterghem I, Rinke C, Parks DH, Zhang Y, Takai K, Sievert SM, Simon J, Campbell BJ, Hanson TE, Woyke T, Klotz MG, Hugenholtz P. 2017. Comparative genomic analysis of the class *Epsilonproteobacteria* and proposed reclassification to Epsilonbacteraeota (phyl. nov.). Front Microbiol 8:682. doi:10.3389/fmicb.2017.00682. 28484436 PMC5401914

[60] Villalobo A, Roldan JM, Rivas J, Cárdenas J. 1977. Assimilatory nitrate reductase from Acinetobacter calcoaceticus. Arch Microbiol 112:127–132. doi:10.1007/BF00429324. 849099

[61] Sievert SM, Wieringa EBA, Wirsen CO, Taylor CD. 2007. Growth and mechanism of filamentous-sulfur formation by Candidatus Arcobacter sulfidicus in opposing oxygen-sulfide gradients. Environ Microbiol 9:271–276. doi:10.1111/j.1462-2920.2006.01156.x. 17227432

[62] Stampi S, Varoli O, Zanetti F, De Luca G. 1993. *Arcobacter cryaerophilus* and thermophilic campylobacters in a sewage treatment plant in Italy: two secondary treatments compared. Epidemiol Infect 110:633–639. doi:10.1017/S0950268800051050. 8519328 PMC2272278

[63] Sasi Jyothsna TS, Rahul K, Ramaprasad EVV, Sasikala C, Ramana CV. 2013. Arcobacter anaerophilus sp. nov., isolated from an estuarine sediment and emended description of the genus Arcobacter. Int J Syst Evol Microbiol 63:4619–4625. doi:10.1099/ijs.0.054155-0. 23918794

[64] Pérez-Cataluña A, Salas-Massó N, Diéguez AL, Balboa S, Lema A, Romalde JL, Figueras MJ. 2018. Revisiting the taxonomy of the genus arcobacter: getting order from the chaos. Front Microbiol 9:2077. doi:10.3389/fmicb.2018.02077. 30233547 PMC6131481

[65] Daughton CG. 2020. Wastewater surveillance for population-wide Covid-19: The present and future. Sci Total Environ 736:139631. doi:10.1016/j.scitotenv.2020.139631. 32474280 PMC7245244

[66] Fernandez-Cassi X, Timoneda N, Martínez-Puchol S, Rusiñol M, Rodriguez-Manzano J, Figuerola N, Bofill-Mas S, Abril JF, Girones R. 2018. Metagenomics for the study of viruses in urban sewage as a tool for public health surveillance. Sci Total Environ 618:870–880. doi:10.1016/j.scitotenv.2017.08.249. 29108696

[67] Hendriksen RS, Munk P, Njage P, van Bunnik B, McNally L, Lukjancenko O, Röder T, Nieuwenhuijse D, Pedersen SK, Kjeldgaard J, Kaas RS, Clausen PTLC, Vogt JK, Leekitcharoenphon P, van de Schans MGM, Zuidema T, de Roda Husman AM, Rasmussen S, Petersen B, Global Sewage Surveillance project consortium, Amid C, Cochrane G, Sicheritz-Ponten T, Schmitt H, Alvarez JRM, Aidara-Kane A, Pamp SJ, Lund O, Hald T, Woolhouse M, Koopmans MP, Vigre H, Petersen TN, Aarestrup FM. 2019. Global monitoring of antimicrobial resistance based on metagenomics analyses of urban sewage. Nat Commun 10:1124. doi:10.1038/s41467-019-08853-3. 30850636 PMC6408512

[68] Aarestrup FM, Woolhouse MEJ. 2020. Using sewage for surveillance of antimicrobial resistance. Science 367:630–632. doi:10.1126/science.aba3432. 32029617

[69] Feng S, McLellan SL. 2019. Highly specific sewage-derived bacteroides quantitative PCR assays target sewage-polluted waters. Appl Environ Microbiol 85:1–15. doi:10.1128/AEM.02696-18. PMC6414369 30635376

[70] Arumugam M, Raes J, Pelletier E, Le Paslier D, Yamada T, Mende DR, Fernandes GR, Tap J, Bruls T, Batto J-M, Bertalan M, Borruel N, Casellas F, Fernandez L, Gautier L, Hansen T, Hattori M, Hayashi T, Kleerebezem M, Kurokawa K, Leclerc M, Levenez F, Manichanh C, Nielsen HB, Nielsen T, Pons N, Poulain J, Qin J, Sicheritz-Ponten T, Tims S, Torrents D, Ugarte E, Zoetendal EG, Wang J, Guarner F, Pedersen O, de Vos WM, Brunak S, Doré J, Antolín M, Artiguenave F, Blottiere HM, Almeida M, Brechot C, Cara C, Chervaux C, Cultrone A, Delorme C, Denariaz G, Dervyn R, MetaHIT Consortium, et al. 2011. Enterotypes of the human gut microbiome. Nature 473:174–180. doi:10.1038/nature09944. 21508958 PMC3728647

[71] Doi Y, Iovleva A, Bonomo RA. 2017. The ecology of extended-spectrum β-lactamases (ESBLs) in the developed world. J Travel Med 24:S44–S51. doi:10.1093/jtm/taw102. 28521000 PMC5731446

[72] Green HC, Haugland RA, Varma M, Millen HT, Borchardt MA, Field KG, Walters WA, Knight R, Sivaganesan M, Kelty CA, Shanks C. 2014. Improved HF183 quantitative real-time PCR assay for characterization of human fecal pollution in ambient surface water. Appl Environ Microbiol 80:3086–3094. doi:10.1128/AEM.04137-13. 24610857 PMC4018914

[73] Feng S, Bootsma M, McLellan SL. 2018. Human-associated Lachnospiraceae genetic markers improve detection of fecal pollution sources in urban waters. Appl Environ Microbiol 84:e00309-18. doi:10.1128/AEM.00309-18. 29728386 PMC6029095

[74] Collado L, Figueras MJ. 2011. Taxonomy, epidemiology, and clinical relevance of the genus *Arcobacter*. Clin Microbiol Rev 24:174–192. doi:10.1128/CMR.00034-10. 21233511 PMC3021208

[75] Al Atrouni A, Joly-Guillou ML, Hamze M, Kempf M. 2016. Reservoirs of non-baumannii Acinetobacter species. Front Microbiol 7:1. doi:10.3389/fmicb.2016.00049. 26870013 PMC4740782

[76] Lindner BG, Suttner B, Zhu KJ, Conrad RE, Rodriguez-R LM, Hatt JK, Brown J, Konstantinidis KT. 2022. Toward shotgun metagenomic approaches for microbial source tracking sewage spills based on laboratory mesocosms. Water Res 210:117993. doi:10.1016/j.watres.2021.117993. 34979467

[77] Cui Q, Huang Y, Wang H, Fang T. 2019. Diversity and abundance of bacterial pathogens in urban rivers impacted by domestic sewage. Environ Pollut 249:24–35. doi:10.1016/j.envpol.2019.02.094. 30877966

[78] Carney RL, Labbate M, Siboni N, Tagg KA, Mitrovic SM, Seymour JR. 2019. Urban beaches are environmental hotspots for antibiotic resistance following rainfall. Water Res 167:115081. doi:10.1016/j.watres.2019.115081. 31574348

[79] Kristensen JM, Nierychlo M, Albertsen M, Nielsen PH, Zhou N-Y. 2020. Bacteria from the genus *Arcobacter* are abundant in effluent from wastewater treatment plants. Appl Environ Microbiol 86:e03044-19. doi:10.1128/AEM.03044-19. 32111585 PMC7170470

[80] Newton RJ, Bootsma MJ, Morrison HG, Sogin ML, McLellan SL. 2013. A microbial signature approach to identify fecal pollution in the waters off an urbanized coast of Lake Michigan. Microb Ecol 65:1011–1023. doi:10.1007/s00248-013-0200-9. 23475306 PMC4084971

[81] Fresia P, Antelo V, Salazar C, Giménez M, D’Alessandro B, Afshinnekoo E, Mason C, Gonnet GH, Iraola G. 2019. Urban metagenomics uncover antibiotic resistance reservoirs in coastal beach and sewage waters. Microbiome 7:35. doi:10.1186/s40168-019-0648-z. 30819245 PMC6396544

[82] Gwenzi W, Musiyiwa K, Mangori L. 2020. Sources, behaviour and health risks of antimicrobial resistance genes in wastewaters: a hotspot reservoir. J Environ Chem Eng 8:102220. doi:10.1016/j.jece.2018.02.028.

[83] Choi PM, Tscharke BJ, Donner E, O'Brien JW, Grant SC, Kaserzon SL, Mackie R, O'Malley E, Crosbie ND, Thomas KV, Mueller JF. 2018. Wastewater-based epidemiology biomarkers: past, present and future. Trends Anal Chem 105:453–469. doi:10.1016/j.trac.2018.06.004.

[84] Manchanda V, Sinha S, Singh N. 2010. Multidrug resistant Acinetobacter. J Glob Infect Dis 2:291–304. doi:10.4103/0974-777X.68538. 20927292 PMC2946687

[85] Alonso A, Sánchez P, Martínez JL. 2001. Environmental selection of antibiotic resistance genes. Environ Microbiol 3:1–9. doi:10.1046/j.1462-2920.2001.00161.x. 11225718

[86] Ju F, Beck K, Yin X, Maccagnan A, McArdell CS, Singer HP, Johnson DR, Zhang T, Bürgmann H. 2019. Wastewater treatment plant resistomes are shaped by bacterial composition, genetic exchange, and upregulated expression in the effluent microbiomes. ISME J 13:346–360. doi:10.1038/s41396-018-0277-8. 30250051 PMC6331547

[87] Peccia J, Zulli A, Brackney DE, Grubaugh ND, Kaplan EH, Casanovas-Massana A, Ko AI, Malik AA, Wang D, Wang M, Warren JL, Weinberger DM, Arnold W, Omer SB. 2020. Measurement of SARS-CoV-2 RNA in wastewater tracks community infection dynamics. Nat Biotechnol 38:1164–1167. doi:10.1038/s41587-020-0684-z. 32948856 PMC8325066

[88] Strange JES, Leekitcharoenphon P, Møller FD, Aarestrup FM. 2021. Metagenomics analysis of bacteriophages and antimicrobial resistance from global urban sewage. Sci Rep 11:1600. doi:10.1038/s41598-021-80990-6. 33452346 PMC7810828

[89] Morrison HG, Grim SL, Vineis JH, Sogin ML. 2013. 16S amplicon Illumina sequencing methods. Figshare. doi:10.6084/m9.figshare.833944.v1.

[90] Huse SM, Welch DBM, Voorhis A, Shipunova A, Morrison HG, Eren AM, Sogin ML. 2014. VAMPS: A website for visualization and analysis of microbial population structures. BMC Bioinformatics 15:41. Accessed, June 2014. doi:10.1186/1471-2105-15-41. 24499292 PMC3922339

[91] Eren AM, Morrison HG, Lescault PJ, Reveillaud J, Vineis JH, Sogin ML. 2015. Minimum entropy decomposition: unsupervised oligotyping for sensitive partitioning of high-throughput marker gene sequences. ISME J 9:968–979. doi:10.1038/ismej.2014.195. 25325381 PMC4817710

[92] Wang Q, Garrity GM, Tiedje JM, Cole JR. 2007. Naïve Bayesian classifier for rapid assignment of rRNA sequences into the new bacterial taxonomy. Appl Environ Microbiol 73:5261–5267. doi:10.1128/AEM.00062-07. 17586664 PMC1950982

[93] Eren AM, Vineis JH, Morrison HG, Sogin ML. 2013. A filtering method to generate high quality short reads using illumina paired-end technology. PLoS One 8:e66643. doi:10.1371/journal.pone.0066643. 23799126 PMC3684618

[94] Li D, Liu CM, Luo R, Sadakane K, Lam TW. 2015. MEGAHIT: an ultra-fast single-node solution for large and complex metagenomics assembly via succinct de Bruijn graph. Bioinformatics 31:1674–1676. doi:10.1093/bioinformatics/btv033. 25609793

[95] Delmont TO, Quince C, Shaiber A, Esen ÖC, Lee ST, Rappé MS, McLellan SL, Lücker S, Eren AM. 2018. Nitrogen-fixing populations of Planctomycetes and Proteobacteria are abundant in surface ocean metagenomes. Nat Microbiol 3:804–813. doi:10.1038/s41564-018-0176-9. 29891866 PMC6792437

[96] Eren AM, Esen OC, Quince C, Vineis JH, Morrison HG, Sogin ML, Delmont TO. 2015. Anvi’o: an advanced analysis and visualization platformfor ’omics data. PeerJ 3:e1319-29. doi:10.7717/peerj.1319. 26500826 PMC4614810

[97] Pritchard L, Glover RH, Humphris S, Elphinstone JG, Toth IK. 2016. Genomics and taxonomy in diagnostics for food security: soft-rotting enterobacterial plant pathogens. Anal Methods 8:12–24. doi:10.1039/C5AY02550H.

[98] Langmead B, Salzberg SL. 2012. Fast gapped-read alignment with Bowtie 2. Nat Methods 9:357–359. doi:10.1038/nmeth.1923. 22388286 PMC3322381

[99] Arango-Argoty G, Garner E, Pruden A, Heath LS, Vikesland P, Zhang L. 2018. DeepARG: a deep learning approach for predicting antibiotic resistance genes from metagenomic data. Microbiome 6:1–15. doi:10.1186/s40168-018-0401-z. 29391044 PMC5796597

[100] Li H. 2011. A statistical framework for SNP calling, mutation discovery, association mapping and population genetical parameter estimation from sequencing data. Bioinformatics 27:2987–2993. doi:10.1093/bioinformatics/btr509. 21903627 PMC3198575

[101] Camacho C, Coulouris G, Avagyan V, Ma N, Papadopoulos J, Bealer K, Madden TL. 2009. BLAST+: architecture and applications. BMC Bioinformatics 10:9. doi:10.1186/1471-2105-10-421. 20003500 PMC2803857

[102] Caporaso JG, Lauber CL, Walters WA, Berg-Lyons D, Lozupone CA, Turnbaugh PJ, Fierer N, Knight R. 2011. Global patterns of 16S rRNA diversity at a depth of millions of sequences per sample. Proc Natl Acad Sci USA 108:4516–4522. doi:10.1073/pnas.1000080107. 20534432 PMC3063599

[103] Martin M. 2011. Cutadapt removes adapter sequences from high-throughput sequencing reads. EMBnetjournal 17:10–12. doi:10.14806/ej.17.1.200.

[104] Deng Y, Jiang YH, Yang Y, He Z, Luo F, Zhou J. 2012. Molecular ecological network analyses. BMC Bioinformatics 13:113. doi:10.1186/1471-2105-13-113. 22646978 PMC3428680

[105] Newman MEJ. 2004. Fast algorithm for detecting community structure in networks. Phys Rev E Stat Nonlin Soft Matter Phys 69:066133. doi:10.1103/PhysRevE.69.066133. 15244693

[106] Guimerà R, Nunes Amaral LA. 2005. Functional cartography of complex metabolic networks. Nature 433:895–900. doi:10.1038/nature03288. 15729348 PMC2175124

[107] Shannon P, Markiel A, Ozier O, Baliga NS, Wang JT, Ramage D, Amin N, Schwikowski B, Ideker T. 2003. Cytoscape: a software environment for integrated models of biomolecular interaction networks. Genome Res 13:2498–2504. doi:10.1101/gr.1239303. 14597658 PMC403769

[108] R Core Team. 2020. R: a language and environment for statistical computing. R Foundation for Statistical Computing, Vienna, Austria.

[109] Peres-Neto PR, Legendre P, Dray S, Borcard D. 2006. Variation partitioning of species data matrices: estimation and comparison of fractions. Ecology 87:2614–2625. doi:10.1890/0012-9658(2006)87[2614:VPOSDM]2.0.CO;2. 17089669

[110] Oksanen J, Blanchet FG, Friendly M, Kindt R, Legendre P, McGlinn D, Minchin RP, O’Hara RB, Simpson GL, Solymos P, Stevens MH, Szoecs E, Wagner H. 2019. Vegan: community ecology package. R Package version 25-5.

[111] Liaw A, Wiener M. 2002. Classification and regression by randomForest. R News 2:18–22. Version 4.6-14. Accessed December 17, 2019.

[112] Dasgupta A. 2014. reprtree: representative trees from ensembles. R package version 0.6. Accessed December 17, 2019. https://github.com/araastat/reprtree.

