# Supplementary figures and images for: Guts of the Urban Ecosystem: Microbial Ecology of Sewer Infrastructure
Source: mSystems. 2022 Jun 28;7(4):e00118-22. doi: 10.1128/msystems.00118-22 (PMC9426572; doi:10.1128/msystems.00118-22)

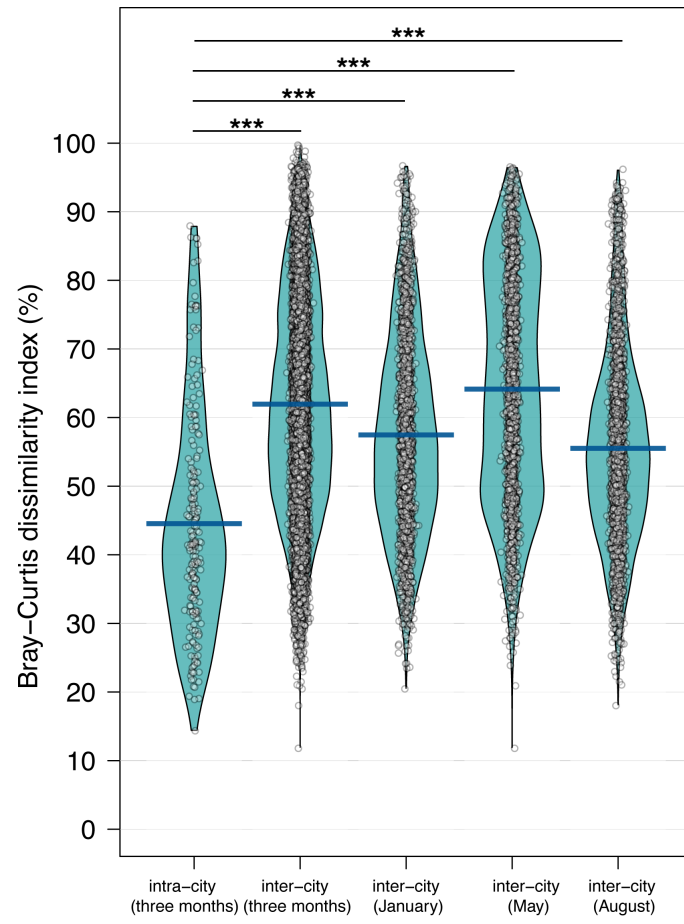

Supplement: FIG S1 [file msystems.00118-22-sf001.pdf]

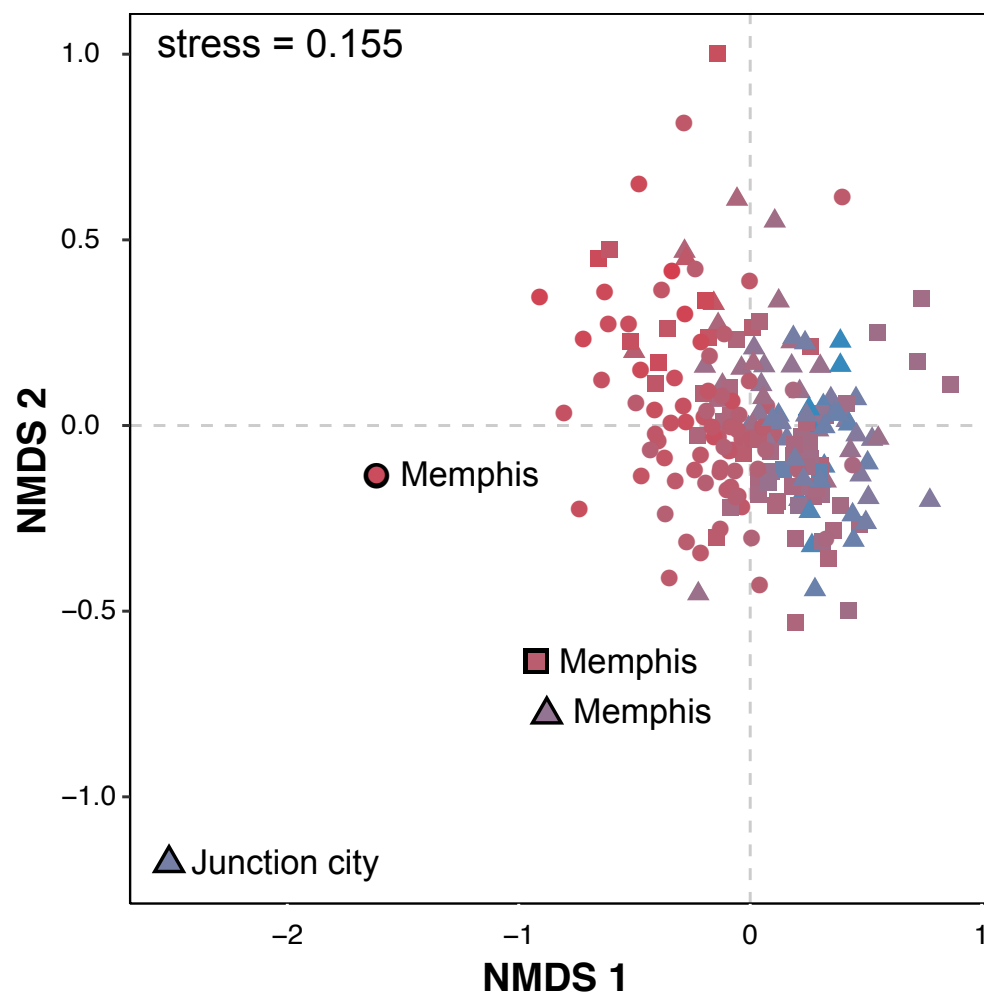

Supplement: FIG S2 [file msystems.00118-22-sf002.pdf]

**a**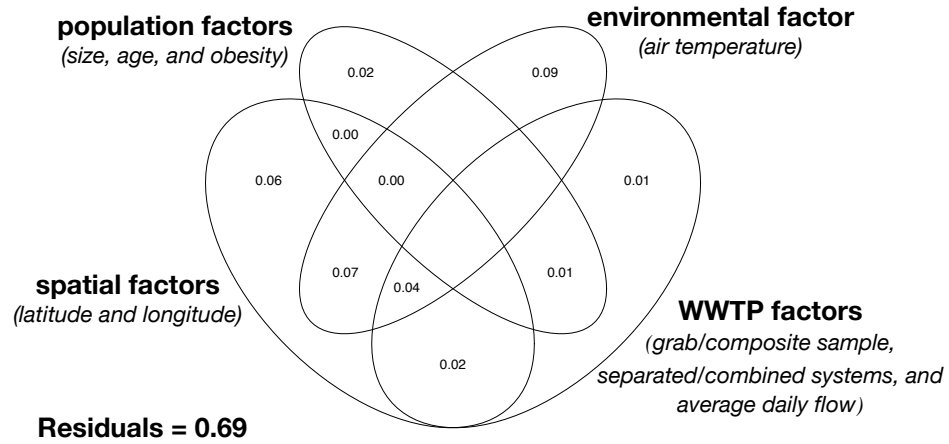**b**

|                 | Degrees of freedom | Variance | F       | P-value (>F) |
|-----------------|--------------------|----------|---------|--------------|
| latitude        | 1                  | 0.021021 | 17.3023 | 0.001***     |
| longitude       | 1                  | 0.004253 | 3.5009  | 0.008**      |
| air temperature | 1                  | 0.027981 | 23.0313 | 0.001***     |
| Residual        | 180                | 0.218682 |         |              |

Supplement: FIG S3 [file msystems.00118-22-sf003.pdf]

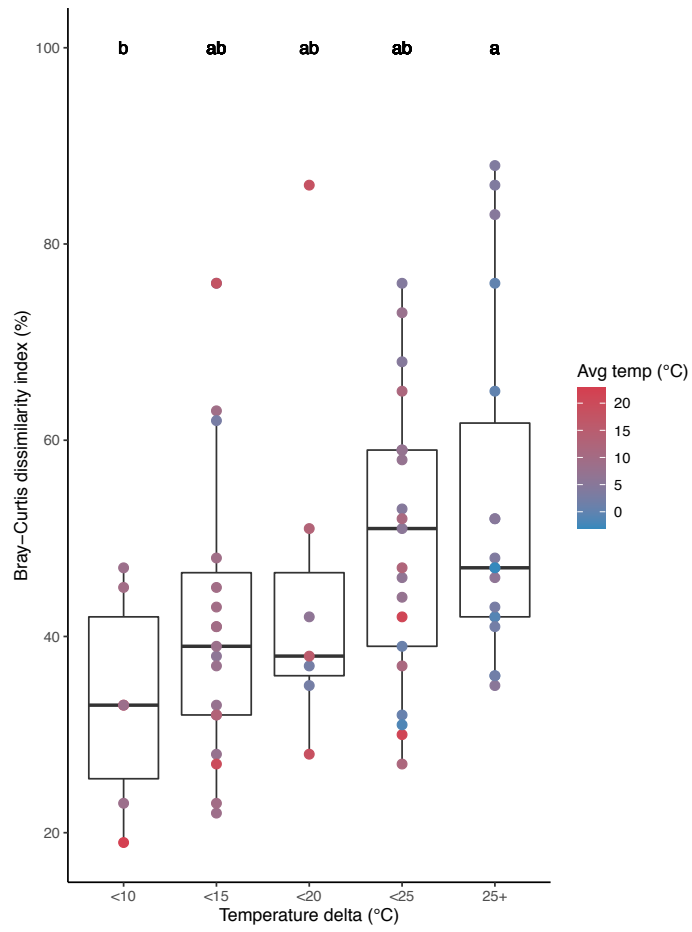

Supplement: FIG S4 [file msystems.00118-22-sf004.pdf]

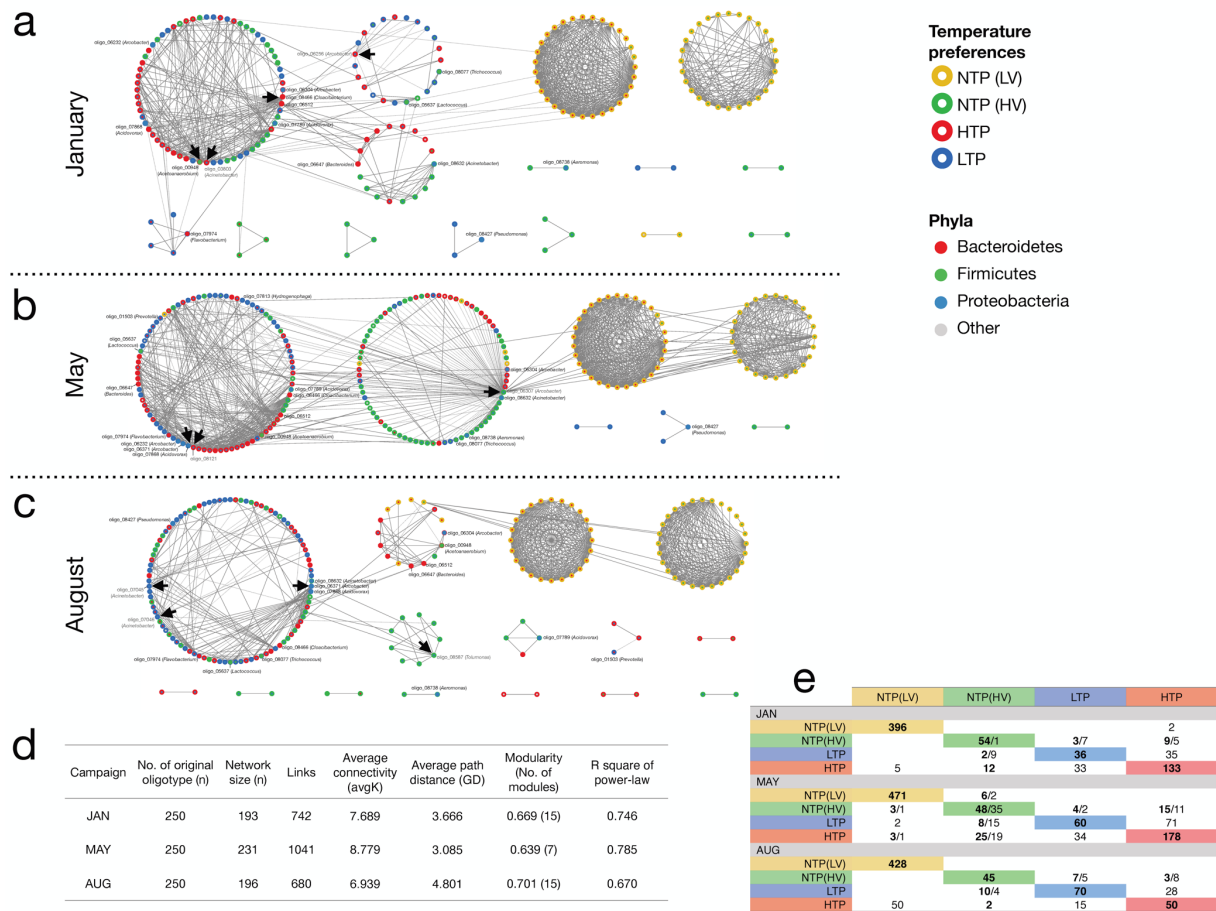

Supplement: FIG S5 [file msystems.00118-22-sf005.pdf]

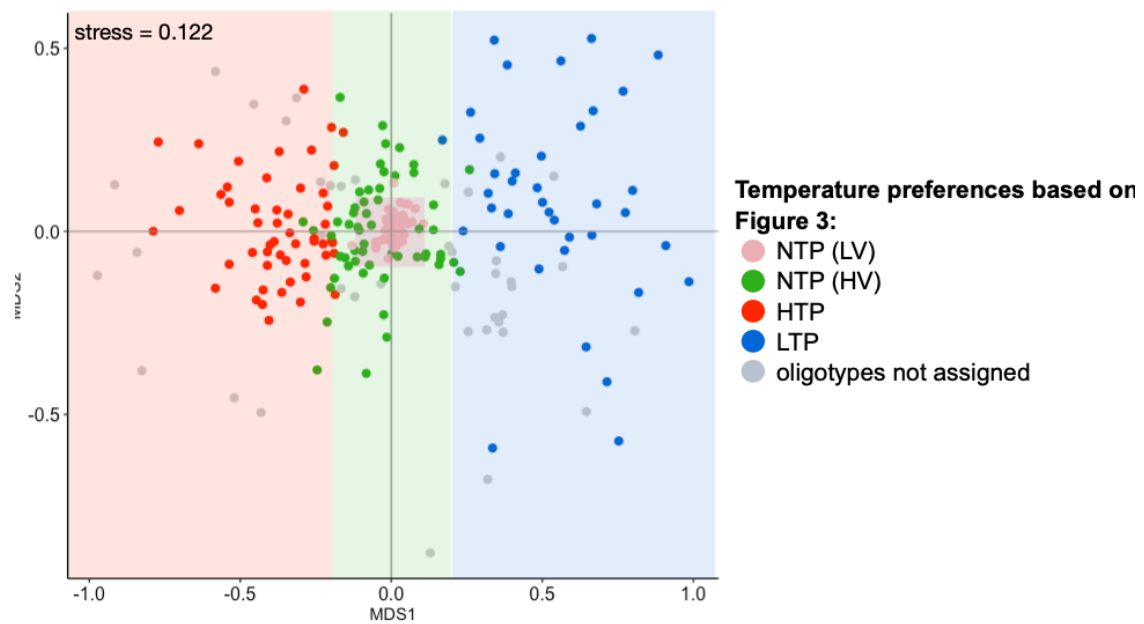

Supplement: FIG S7 [file msystems.00118-22-sf007.pdf]

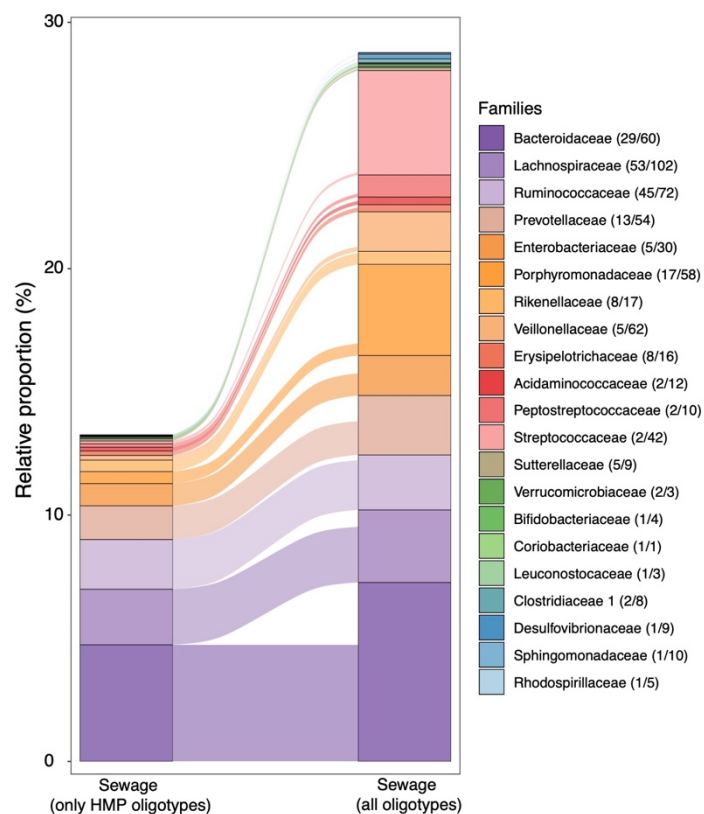

Supplement: FIG S6 [file msystems.00118-22-sf006.pdf]
